# Comparison of Two Approaches for the Metataxonomic Analysis of the Human Milk Microbiome

**DOI:** 10.3389/fcimb.2021.622550

**Published:** 2021-03-25

**Authors:** Lorena Ruiz, Claudio Alba, Cristina García-Carral, Esther A. Jiménez, Kimberly A. Lackey, Michelle K. McGuire, Courtney L. Meehan, James Foster, Daniel W. Sellen, Elizabeth W. Kamau-Mbuthia, Egidioh W. Kamundia, Samwel Mbugua, Sophie E. Moore, Andrew M. Prentice, Debela Gindola K, Gloria E. Otoo, Rossina G. Pareja, Lars Bode, Mark A. McGuire, Janet E. Williams, Juan M. Rodríguez

**Affiliations:** ^1^ Department of Nutrition and Food Science, Complutense University of Madrid, Madrid, Spain; ^2^ Margaret Ritchie School of Family and Consumer Sciences, University of Idaho, Moscow, ID, United States; ^3^ Department of Anthropology, Washington State University, Pullman, WA, United States; ^4^ Dalla Lana School of Public Health, University of Toronto, Toronto, ON, Canada; ^5^ Department of Human Nutrition, Egerton University, Nakuru, Kenya; ^6^ Division of Women’s Health, King’s College London, London, United Kingdom; ^7^ MRC Unit, Serekunda, Gambia; ^8^ MRC International Nutrition Group, London School of Hygiene and Tropical Medicine, London, United Kingdom; ^9^ Department of Anthropology, Hawassa University, Hawassa, Ethiopia; ^10^ Department of Nutrition and Food Science, University of Ghana, Accra, Ghana; ^11^ Instituto de Investigación Nutricional, Lima, Peru; ^12^ Department of Pediatrics and Larsson-Rosenquist Foundation Mother-Milk-Infant Center of Research Excellence (MOMI CoRE), University of California, San Diego, La Jolla, CA, United States; ^13^ Department of Animal and Veterinary Science, University of Idaho, Moscow, ID, United States

**Keywords:** human milk, microbiota, 16S rRNA, bacteria, sequencing reproducibility

## Abstract

Recent work has demonstrated the existence of large inter-individual and inter-population variability in the microbiota of human milk from healthy women living across variable geographical and socio-cultural settings. However, no studies have evaluated the impact that variable sequencing approaches targeting different 16S rRNA variable regions may have on the human milk microbiota profiling results. This hampers our ability to make meaningful comparisons across studies. In this context, the main purpose of the present study was to re-process and re-sequence the microbiome in a large set of human milk samples (n = 412) collected from healthy women living at diverse international sites (Spain, Sweden, Peru, United States, Ethiopia, Gambia, Ghana and Kenya), by targeting a different 16S rRNA variable region and reaching a larger sequencing depth. Despite some differences between the results obtained from both sequencing approaches were notable (especially regarding alpha and beta diversities and Proteobacteria representation), results indicate that both sequencing approaches revealed a relatively consistent microbiota configurations in the studied cohorts. Our data expand upon the milk microbiota results we previously reported from the INSPIRE cohort and provide, for the first time across globally diverse populations, evidence of the impact that different DNA processing and sequencing approaches have on the microbiota profiles obtained for human milk samples. Overall, our results corroborate some similarities regarding the microbial communities previously reported for the INSPIRE cohort, but some differences were also detected. Understanding the impact of different sequencing approaches on human milk microbiota profiles is essential to enable meaningful comparisons across studies.

**Clinical Trial Registration:**

www.clinicaltrials.gov, identifier NCT02670278.

## Introduction

Despite the fact that human milk has long been considered sterile, research conducted over the last decade has provided convincing evidence that this biological fluid harbors a rich microbial community under all physiological circumstances (Martín et al., 2006; Martín et al., 2007; Solis et al., 2010; Fernández et al., 2013). Milk’s microbial community contains an important arsenal of bacterial and fungal species of substantial interest as they likely play crucial roles in the maintenance of maternal and infant health (as reviewed in Boix-Amorós et al., 2019; Moossavi et al., 2020; Stinson et al., 2020). For instance, they likely seed the breastfed infant’s gastrointestinal (GI) tract, initiating the assembly of a mature healthy human GI microbiota, and orchestrating the innate immunity maturation and programming that will condition infant health outcomes in the short and long term (as reviewed in Milani et al., 2017). The milk microbiota in conjunction with other bioactive factors present in human milk, such as oligosaccharides and immune factors, have been deemed responsible for many of the long-term, health promoting effects associated with exclusive breastfeeding in early life, which correlate with reduced incidence of chronic inflammatory and metabolic conditions in infancy and adulthood (Huërou-Luron et al., 2010; Roger et al., 2010; Musilova et al., 2017).

Indeed, a growing literature supporting the crucial roles exerted by the human milk microbiota in maternal and infant health, in conjunction with advances in high-throughput sequencing (HTS) technologies, have led to a rapidly growing interest in the study of this microbial community and its variation, mainly in relation to maternal and infant factors. In this regard evidence suggests the existence of a strong inter-individual variation in the composition of the human milk microbiota across different populations, in relation to variable delivery factors (Cabrera-Rubio et al., 2016; Hoashi et al., 2016; Asbury et al., 2020), lactation stage (Khodayar-Pardo et al., 2014), maternal conditions (either chronic pre-pregnancy situations or those developed during pregnancy) (LeMay-Nedjelski et al., 2020; Volery et al., 2020; Wan et al., 2020), lifestyle habits (Moossavi et al., 2019; Padilha et al., 2020), psycho-social and economic conditions (Ojo-Okunola et al., 2019), and infant health outcomes (Demmelmair et al., 2020) as summarized previously in Ruiz et al. (2019) and Lackey et al. (2019). More recently, a combination of HTS and culturomic approaches has further supported the existence of a diversity of viable bacterial cells in healthy human milk wider than previously anticipated, and has offered novel opportunities to conduct mechanistic studies on the metabolic potential of this microbial community (Schwab et al., 2019; Togo et al., 2019; Treven et al., 2019).

While most investigators have undertaken research on this field with the aim to identify imbalances or dysbiosis states under specific maternal/infant conditions or in relation to health outcomes, very little effort has been aimed at delineating the structure of a healthy human milk microbiota, even though defining the normal baseline of a given microbiota is essential for a comprehensive understanding into the variation associated with different health outcomes (Bäckhed et al., 2012). Moreover, early studies on the structure of the healthy human milk microbiota conducted on either US (Hunt et al., 2011), Finnish (Cabrera-Rubio et al., 2012), Mexican-American (Davé et al., 2016), Chinese (Li et al., 2017), and Canadian populations (Moossavi et al., 2019), suggested the existence of significant variation across populations. For instance while most studies concur regarding the identification of a few “core” and dominant bacterial genera including mainly *Staphylococcus* and *Streptococcus* species, other representative microbial organisms belonging to the lactic acid bacteria group (*Lactobacillus, Lactococcus, Leuconostoc, Weisella*), or typical skin inhabitants such as *Propionibacterium* (*Cutibacterium*) or *Corynebacterium* are not universally detected across all populations analyzed despite appearing as predominant in some. For instance, *Lactococcus, Leuconostoc*, and *Weisella* appear to be dominant taxa in Finnish women (Cabrera-Rubio et al., 2012), while in Chinese and Taiwanese women the dominant populations included *Pseudomonadaceae* and *Lactobacillaceae* (Li et al., 2017). In addition, some research such as that reported by Moosssavi and colleagues, have identified different milk biome “types” even within the same Canadian cohort, supporting the existence of intrapopulation variability (Moossavi et al., 2019). However, most of these studies have been conducted on a limited number of samples or population groups, and have employed non-standardized procedures which could have introduced important biases in the microbiota profiling (Panek et al., 2018). These facts have impeded our ability to make meaningful comparisons across studies and prevented our ability to understand genuine biological variation in the microbiota inherent in milk produced by healthy women across populations.

It is also worth remarking that, whereas some efforts to achieve standardization of sample processing and analysis in the context of the human GI microbiota have been reported as recently reviewed (Wu et al., 2019), such initiatives have not yet been tackled in the context of the human milk microbiota which, due to its intrinsic physiological and microbiological characteristics (Moossavi et al., 2019), might be strongly affected by variable collection and analytical processing.

We recently attempted to fill these knowledge gaps by reporting a large, cross-sectional study on the healthy human milk microbiome across a cohort of over 400 healthy lactating women from selected geographically diverse populations living across three different continents, by using standardized sample collection and processing approaches (Lackey et al., 2019). As expected, we found substantial variation in the milk microbiome among cohorts. In the present work, we provide even more insight into the impact that variable sample processing and data analysis pipelines might have on the study and interpretation of the milk microbiota landscape, through extraction and sequencing the same set of milk samples using an amplicon approach targeting a different 16S rRNA variable region with greater sequencing depth, and performing a comparative analysis with the previously reported dataset.

## Materials and Methods

### Design, Setting, and Sampling

The design of the cross-sectional, epidemiological, multi-cohort study has been described in detail (McGuire et al., 2017; Ruiz et al., 2017; Lackey et al., 2019; Lane et al., 2019). All study procedures were approved by the overarching Washington State University Institutional Review Board (#13264) and at each study location, and consent was obtained from each participating woman. Milk samples from 412 mothers were obtained from 11 different populations, including one cohort from Kenya (KE) (n=42), Ghana (GN) (n=40), Peru (PE) (n=43), Sweden (SW) (n=24), Spain (SP) (n=41), and two cohorts from Ethiopia (rural [ETR] [n=40]; and urban [ETU] [n=40], cohorts), two from Gambia (rural [GBR] [n=40] and urban [GBU] [n=40], cohorts), and two from the United States (San Diego, California [USC] [n=19] and Washington/Idaho [USW] [n=41] cohorts) (**Supplementary Figure 1**). Milk was collected as described previously (McGuire et al., 2017). Briefly, following skin cleaning twice with single use and using gloved hands, milk was manually expressed and collected into disposable sterile containers, with the exception of milk samples from USC, USW, SW and PE cohorts, which were pump-expressed by using an electric pump and sterile disposable containers. Milk from each woman was aliquoted into two samples for metataxonomic analysis; one was shipped on dry ice to the University of Idaho (USA), and the second was shipped on dry ice to the Complutense University of Madrid (Spain); in both locations the samples were immediately frozen at −20°C. Due to unreliable access to electricity supply and/or freezers, milk samples collected from the ETR cohort were immediately mixed at a 1:1 ratio with Milk Preservation Solution (Norgen Biotek, Ontario) and frozen within 6 days as it has previously been demonstrated capable to preserve bacterial DNA integrity for at least two weeks (Lackey et al., 2017). Whereas milk microbiome data garnered from methods utilized at the University of Idaho have been published (Lackey et al., 2019), here we report results from a companion analysis conducted in our laboratory in Spain. We also compare our results to those previously reported using different analytical and bioinformatic approaches.

### DNA Extraction From Milk

For the DNA isolation from all sample cohorts, with the exception of ETR, approximately 1 mL of each sample was used for DNA extraction following the method described by Castro et al. (2019). Briefly, milk samples were thawed on ice and centrifuged (13,000 rpm, 10 min at 4°C), the lipid and supernatant layers were removed and the cell pellet was resuspended in 500 µl TE50 buffer. This solution was further processed for DNA isolation by performing an enzymatic lysis adding 100 µl of an enzymatic mix containing 5 mg/ml of lysozyme (Sigma-Aldrich), 1.5 KU/ml mutanolysin (Sigma-Aldrich) and 120 U/ml lysostaphyn and incubating the samples for 1 hour at 37°C. Subsequently samples were subjected to physical lysis by bead beating with FastPrep Fp120 (Thermo Scientific, Waltham, MA) and glass bead matrix tubes (3 cycles × 60 s, speed 6) in step 4. Finally, DNA was purified by using a modified version of the Qiamp DNA mini kit (Qiagen) columns whereby 100 µl of 3 M sodium acetate pH 5.5 were added to the lysate prior to its addition to the column. Extracted DNA was eluted in 22 μL of nuclease-free water and stored at -20 °C until further analysis. Purity and concentration of each extracted DNA was initially estimated using a NanoDrop 1000 spectrophotometer (NanoDrop Technologies, Inc., Rockland, USA). Negative controls (blanks) were added during the extraction to account for possible contaminants introduced during sample manipulation and DNA isolation. In the particular case of ETR samples, only 250 µl of milk samples were used for DNA isolation since they were the only samples treated with a preservation solution and, under this circumstance, the manufacturer of the preservation solution and companion milk DNA isolation kit recommends not to use a volume larger than 250 µl (Milk DNA Preservation and Isolation Kit, NorgenBiotek, Throlod, Canada).

### Sequencing of Microbial DNA and Bioinformatic Analysis

The V3-V4 hypervariable region of the 16S rRNA was amplified by PCR and sequenced as previously described (Klindworth et al., 2013). Briefly, universal primers S-D-Bact-0341-b-S-17 (CCTACGGGNGGCWGCAG) and S-D-Bact-129 0785-a-A-21 (GACTACHVGGGTATCTAATCC) were used to amplify the V3-V4 hypervariable region of the 16S rRNA and then, barcodes were appended to 3’ and 5’ terminal ends of the PCR amplicons in a second PCR-reaction in order to allow separating forward and reverse sequences. The pooled, purified and barcoded DNA amplicons were sequenced using the Illumina MiSeq 2 x 300 bp paired-end protocol (Illumina Inc., San Diego, CA, USA) following the manufacturer’s recommendations at the facilities of Parque Científico de Madrid (Tres Cantos, Spain) (Klindworth et al., 2013).

Raw sequence data were demultiplexed and quality filtered using Illumina MiSeq Reporter analysis software. Microbiome bioinformatics were performed with QIIME 2 2019.1 (Bolyen et al., 2019). Denoising was performed with DADA2 (Callahan et al., 2016). The forward reads were truncated at position 277 by trimming the last 15 nucleotides, while the reverse ones were truncated at the 250 nucleotides by trimming the last 15 nucleotides, in order to discard positions for which nucleotide median quality were Q20 or below. Samples with less than 1000 sequences (n= 10) were excluded from further analysis.

Taxonomy was assigned to ASVs using the q2-feature-classifier (Bokulich et al., 2018) classify-sklearn naïve Bayes taxonomy classifier against the SILVA 138 reference database (Quast et al., 2013). Subsequent bioinformatic analysis was conducted using R version 3.5.1 (R Core Team, 2013; https://www.R-project.org). The decontam package version 1.2.1 (Davis et al., 2018) was used in order to identify, visualize and remove contaminating DNA with two negative control samples.

A set of “core” genera were characterized for each sample type both in the overall dataset and within each cohort. To be included in the core taxa, a genus must have been represented with a relative abundance higher than 0.1% in, at least, 90% of the samples from one or more cohorts. The 4 most abundant phyla from all the milk samples were selected as most abundant phyla, the rest were included in the “minor_phyla” group and the sequences whose phyla were unknown were grouped in the “unclassified_phyla” group. The 18 most abundant genera from all the milk samples were selected as most abundant genera, the rest were included in the “minor_genera” group and the sequences whose genera were unknown were grouped in the “unclassified_genera” group.

### Comparison of the Results With Those Obtained With the Same Set of Samples but Using a Different Metataxonomic Approach

The results of a previous different metataxonomic approach with the same set of milk samples has been published (Lackey et al., 2019). Methods used for DNA extraction, amplification, assessment of DNA quality, sequencing and statistical analyses are detailed in that publication. Briefly, a dual-barcoded, two-step 30-cycle polymerase chain reaction (PCR) was conducted to amplify the V1-V3 hypervariable region of the 16S rRNA bacterial gene. For the first step, a 7-fold degenerate forward primer targeting position 27 and a reverse primer targeting position 534 (positions numbered according to the *Escherichia coli* rRNA gene) were used as described previously (Carrothers et al., 2015). For the second step, a unique barcoded primer pair with Illumina adaptors attached was added to each sample. Sequences were obtained using an Illumina MiSeq (San Diego, CA) v3 paired-end 300-bp protocol for 600 cycles at the University of Idaho IBEST Genomics Resources Core. However, due to the quality of the ends of the reverse reads, few reads were able to be merged and thus only the forward reads were used in the analyses.

**Figure 1:**
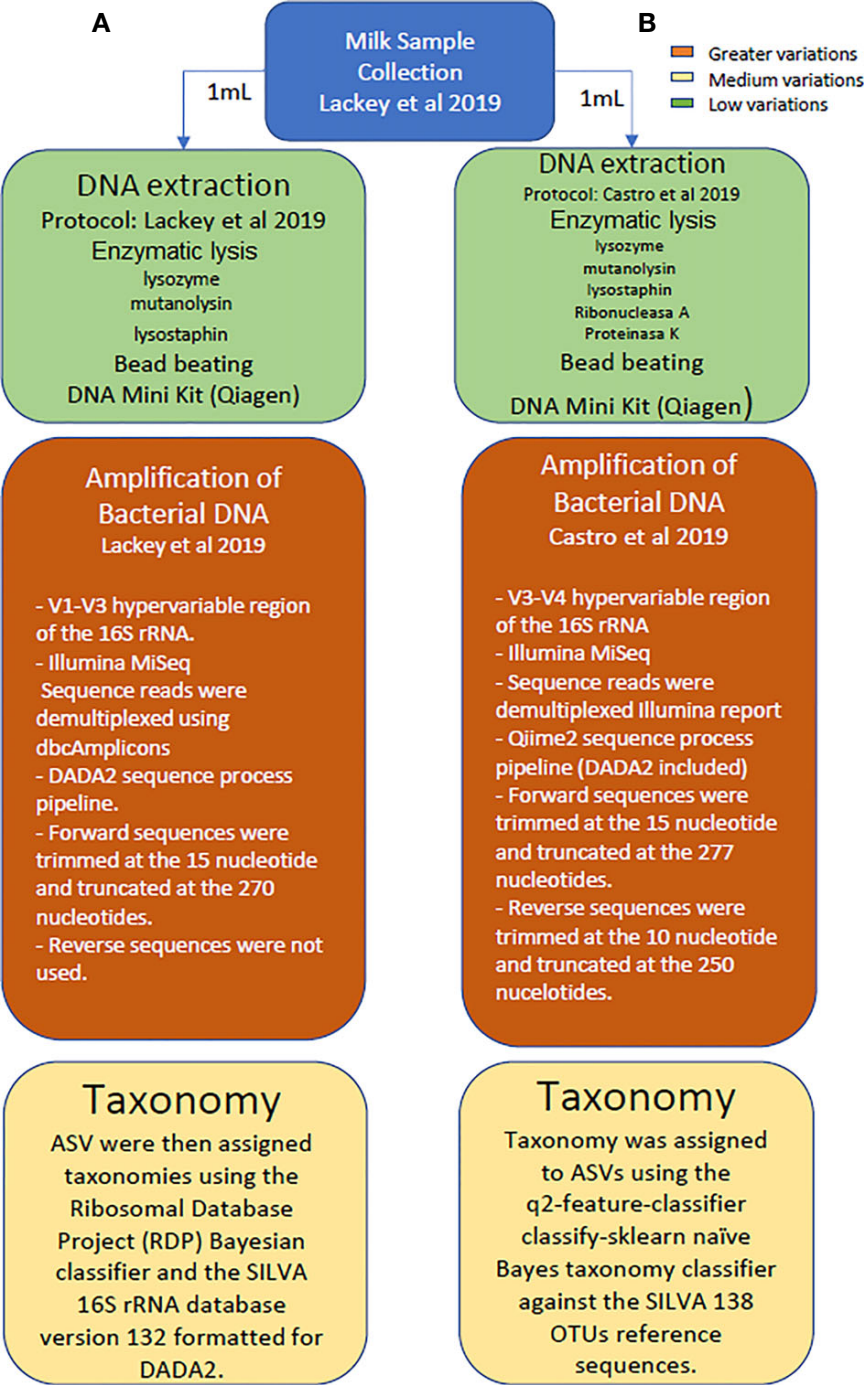
Schematic representation highlighting the main differences in the methodological and analytical strategies followed in the study reported by Lackey and colleagues, as compared to the sequencing approach conducted herein with the same dataset of samples.

A comparison of data obtained from both metataxonomic studies (V1-V3 reported by Lackey and colleagues (2019) versus V3-V4 studies conducted in this work) was made at genus and phylum levels using the SILVA 132 reference database since it was the one used in the first study. A schematic representation highlighting the main differences in the methodological and analytical strategies followed in the study reported by Lackey and colleagues, as compared to the sequencing approach conducted herein with the same dataset of samples is provided in **Figure 1**.

### Statistical Analysis

Quantitative data were expressed as the median and interquartile range (IQR). Differences between groups were assessed using Kruskal-Wallis tests and pairwise Wilcoxon rank sum tests to calculate comparisons between groups. Bonferroni corrections were made to control for multiple comparisons. A table of amplicon sequence variants (ASVs) counts per sample was generated, and bacterial taxa abundances were normalized to the total number of sequences in each sample. Alpha diversity was studied with the Shannon and Simpson diversity indexes with the R vegan package (Version:2.5.6) (Oksanen et al., 2012). Principal coordinates analysis (PCoA) was used to evaluate beta diversity and to plot patterns of bacterial community diversity through a distance matrix containing a dissimilarity value for each pairwise sample comparison. Quantitative (relative abundance) and qualitative (presence/absence) analyses were performed with the Bray-Curtis index and binary Jaccard index, respectively. Analysis of variance of the distance matrices were performed with the “nonparametric MANOVA test” Adonis with 999 permutations as implemented in the R vegan package to reveal statistical significance. For multilevel pairwise Adonis comparisons, the method used for p-value correction was Holm–Bonferroni method with “pairwiseAdonis” R package (version 0.4) (Martínez, 2020). Heatmap hierarchical clustering was performed by using the Euclidean distance and complete hclust_method.

## Results

### Metataxonomic Analysis Targeting the V3-V4 Hypervariable Region of the 16S rRNA Gene With the SILVA 138 Database

The 16S rRNA gene sequencing analysis of the milk samples conducted in this study (*n* = 392) (**Supplementary Figure 1**) yielded 17,622,545 high quality filtered sequences, ranging from 12,981 to 437,280 reads per sample [mean=44,956 reads per sample; median (IQR)=35,754 (26,438-50,539) sequences per sample].

The median values for the Shannon diversity index oscillated between 3.48 (ETR) and 2.44 (USC) while those for the Simpson diversity index ranged between 0.91 (ETR) and 0.93 (GBU) (**Supplementary Table 1**). Overall, there was a significant effect of cohort on both diversity indices (p = 0.001 and p = 0.001, respectively). A comparison at the continent level showed significant differences on both diversity indices when the African cohorts were compared (p<0.002 and p<0.0002, respectively) since samples collected in ETR and GBU exhibited a higher diversity than those obtained in KE. In contrast, no significant differences were found when the different European and US cohorts were compared (p = 0.099 and p = 0.33, respectively) (**Supplementary Table 1**).

**Figure 2:**
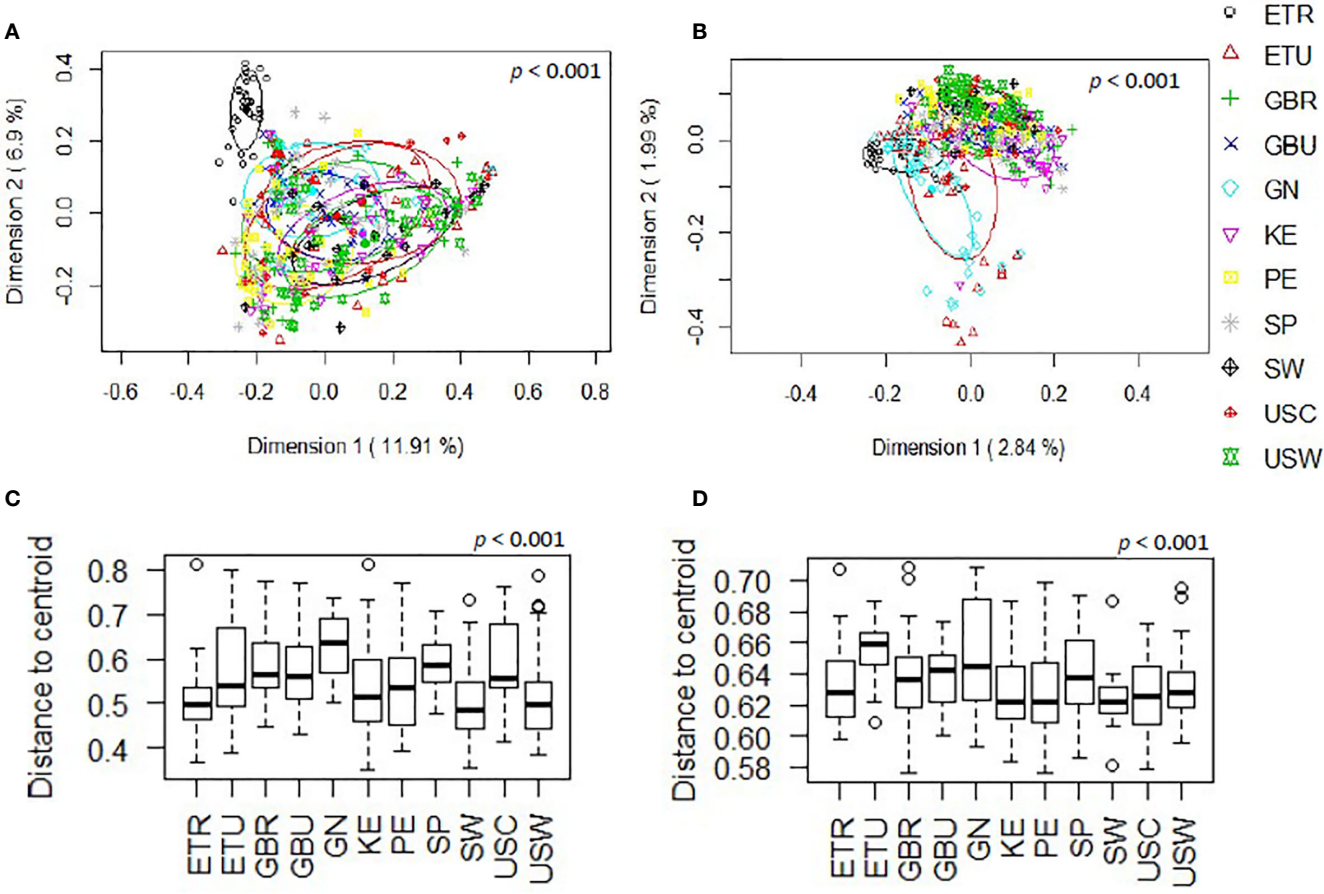
Comparison, at the genus level, of the beta diversity of the different cohorts included in this work. **(A)** Principal coordinate analysis (PCoA) plots of bacterial profiles based on the Bray-Curtis similarity analysis (relative abundance). **(B)** Principal coordinate analysis (PCoA) plots of bacterial profiles at the genus level based on the Jaccard’s coefficient for binary data (presence or absence). The values on each axis label in graphs **(A, B)** represent the percentage of the total variance explained by that axis. The differences between groups of milk samples were analyzed using the PERMANOVA test with 999 permutations. **(C)** Comparison of the mean distances of samples to the centroids in the PCoA plots based on the Bray-Curtis dissimilarity index of each group. **(D)** Comparison of the mean distances of samples to the centroids in the PCoA plots based on the Jaccard’s coefficient of each group.

The overall analysis of the beta diversity, calculated according to the relative abundance of ASVs (Bray-Curtis distance) and the presence/absence of ASVs sequences (binary Jaccard distance matrix), indicated that the profiles of bacterial genera of the different cohorts apparently clustered into different groups (p < 0.001 and p < 0.001, respectively; PERMANOVA) and the ETR cohort was observed to be more clearly separated from the other locations with both distance metrics (**Figures 2A, B**). Besides, when beta diversity of the samples was evaluated according to the continent where the different samples were collected from, the ordination based on relative abundance of ASVs (Bray-Curtis distance) revealed differences between the African and European samples (p < 0.001) and, also, between the African and American samples (p < 0.001). In contrast, differences between the European and American cohorts did not reach statistical significance (p = 0.059). In relation to the analysis of the beta diversity according to the presence/absence of ASVs (binary Jaccard distance matrix), all the comparisons (African *vs.* European samples, African *vs*. American samples, and European *vs.* American samples) revealed the existence of significant differences (p < 0.001). Among African countries all the cohorts showed significant differences for both the relative abundance and the presence/absence of ASVs (p < 0.05). In the European and American countries, only the pairwise comparisons between the SW and USC (for both the relative abundance and the presence/absence); and SW and USW (for relative abundance) showed no statistical differences. The *post-hoc* Holmes-Bonferroni correction of the multilevel pairwise Adonis comparisons increased the *p*-values of most of the previously significant pairwise comparisons from <0.05 to 0.05, with the exception of the comparison between GBU and GBR, which *p*-value changed from 0.04195804 to 0.17 and, therefore, it lost the statistical significance (**Supplementary Table 2**). It must be highlighted that the Holmes-Bonferroni correction involved a high number of comparisons (n=55), rendering it as a very exigent statistical test.

Comparison of the mean distances of samples to the centroids using PCoA plots based either on the Bray-Curtis dissimilarity index or on the Jaccard’s coefficient of each cohort, also revealed the existence of significant differences (p<0.001) (**Figures 2C, D**). Notably, GN was the cohort displaying a higher beta-dispersion, in terms of relative abundance while ETU displayed the highest beta-dispersion in terms of presence/absence of ASVs, suggesting the existence of a greater intrapopulation heterogeneity of microbiota profiles among the samples from these two cohorts, as compared to other populations analyzed in this study; whereas ETR, SW and USC displayed the smallest distances to centroids in terms of relative abundance data, revealing these cohorts present the most homogeneous microbiota profiles.

**Figure 3:**
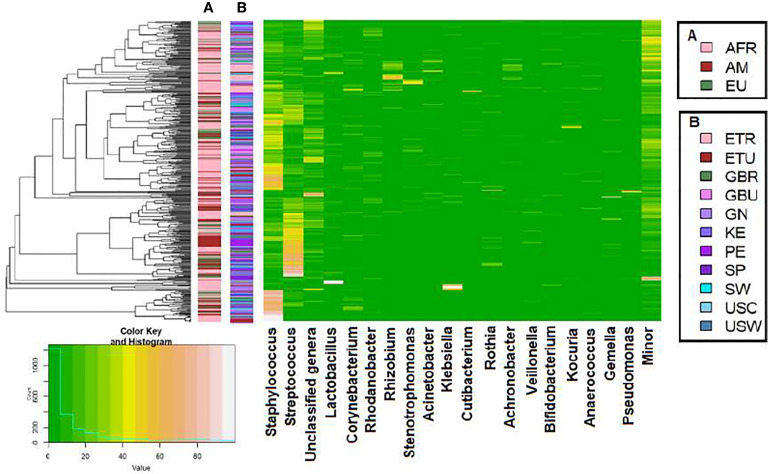
Heatmap plot representing the hierarchical clustering, at the genus level, of the milk samples by continent cohorts **(A)** and by location cohorts **(B)** analyzed in this work with the SILVA 138 database.

A total of 46 phyla were identified in the milk samples, with Firmicutes, Proteobacteria, Actinobacteriota (formerly Actinobacteria) and Patescibacteria being the most abundant. There was a significant effect of cohort on Firmicutes, Proteobacteria, Actinobacteriota, Patescibacteria and the group of “unclassified_phyla” (**Table 1**). Again, the ETR cohort showed greater differences with respect to the rest of the cohorts since it exhibited the lowest relative abundance of Firmicutes and the highest concerning Proteobacteria, Actinobacteriota and Patescibacteria (**Table 1**). An initial assessment of potentially dominant patterns in the bacteriological profile of the milk samples is shown in the heatmap plot presented in **Figure 3**. Overall, there was no clear separation between the milk samples from women of the different cohorts; however, a clear separation could be observed on the basis of the relative abundance of sequences belonging to the genera *Staphylococcus* and *Streptococcus* (**Figure 3**), which seems to be independent of the continent or the cohort. In addition, the clustering analysis suggested that ETR samples separation could be driven by the genera *Rhizobium*, *Achromobacter*, *Corynebacterium* and *Stenotrophomonas* (**Figure 3**). A boxplot of the 9 most abundant genera (including the group of the unclassified ones) found in each location is shown in **Supplementary Figure 2**.

**Table 1:** Relative frequencies, medians and interquartile range (IQR) of the most abundant bacterial phyla (bold) and genera detected in the milk samples analyzed in this work.

Phylum/Genus	ETR		ETU		GBR		GBU		GN		KE		PE		SP		SW		USC		USW		p-value^†^
	n (%)^#^	Median (IQR)	n (%)	Median (IQR)	n (%)	Median (IQR)	n (%)	Median (IQR)	n (%)	Median (IQR)	n (%)	Median (IQR)	n (%)	Median (IQR)	n (%)	Median (IQR)	n (%)	Median (IQR)	n (%)	Median (IQR)	n (%)	Median (IQR)	
**Firmicutes**	32 (100%)	34.13 (21.56-51.3)	40 (100%)	64.65 (36.57-82.26)	40 (100%)	63.34 (43.84-75.13)	39 (97.5%)	53.67 (44.13-62.98)	40 (100%)	37.29 (24.44-71.19)	42 (100%)	69.21 (48.17-74.24)	38 (100%)	73.74 (53.5-85.68)	40 (100%)	52.34 (41.38-80.31)	20 (100%)	63.1 (52.14-80.37)	19 (100%)	72.95 (18.73-83.59)	41 (100%)	60.58 (48.72-70.05)	<0.001
*Staphylococcus*	32 (100%)	9.67 (5.42-16.39)	40 (100%)	29.92 (8.78-48.42)	39 (97.5%)	14.81 (6.96-35.69)	40 (100%)	22.98 (16.06-34.3)	39 (97.5%)	9.59 (3.86-24.52)	42 (100%)	28.49 (13.47-52.45)	36 (94.74%)	18.12 (8.19-31.03)	40 (100%)	14.78 (7.51-33.91)	20 (100%)	26.32 (20.33-49.74)	19 (100%)	10.49 (4.69-51.83)	41 (100%)	25.26 (12.76-45.55)	<0.001
*Streptococcus*	30 (93.75%)	7.3 (3.28-13.29)	35 (87.5%)	5.91 (0.15-16.78)	39 (97.5%)	9.33 (3.59-32.28)	37 (92.5%)	10.25 (4.75-21.88)	36 (90%)	2.49 (0.08-8.88)	40 (95.24%)	15.43 (5.38-29.94)	38 (100%)	37.8 (22.74-57.26)	40 (100%)	12.55 (5.39-52.6)	20 (100%)	11.97 (7.84-28.44)	19 (100%)	11.38 (1.82-31.85)	41 (100%)	15.57 (6.59-28.15)	<0.001
*Lactobacillus*	26 (81.25%)	1.03 (0.25-2.93)	24 (60%)	0.13 (<0.01-0.51)	31 (77.5%)	1.57 (0.06-3.68)	28 (70%)	1.7 (<0.01-5.58)	30 (75%)	1.24 (0.01-5.01)	28 (66.67%)	0.79 (<0.01-2.77)	23 (60.53%)	0.08 (<0.01-1.01)	18 (45%)	<0.01 (<0.01-0.41)	17 (85%)	0.3 (0.05-1.49)	11 (57.89%)	0.02 (<0.01-0.25)	31 (75.61%)	0.44 (0.01-1.28)	<0.001
*Veillonella*	24 (75%)	0.75 (0.04-4.11)	18 (45%)	<0.01 (<0.01-0.28)	24 (60%)	0.3 (<0.01-1.52)	18 (45%)	<0.01 (<0.01-2.31)	12 (30%)	<0.01 (<0.01-0.13)	18 (42.86%)	<0.01 (<0.01-1.18)	27 (71.05%)	1.43 (<0.01-4.08)	18 (45%)	<0.01 (<0.01-0.59)	14 (70%)	0.15 (<0.01-0.72)	12 (63.16%)	0.06 (<0.01-1.54)	22 (53.66%)	0.01 (<0.01-0.6)	<0.001
*Anaerococcus*	23 (71.88%)	1.21 (<0.01-3.15)	21 (52.5%)	0.05 (<0.01-0.38)	24 (60%)	0.14 (<0.01-1.21)	20 (50%)	0.03 (<0.01-1.38)	24 (60%)	0.2 (<0.01-0.53)	18 (42.86%)	<0.01 (<0.01-0.92)	26 (68.42%)	0.3 (<0.01-0.95)	18 (45%)	<0.01 (<0.01-0.78)	13 (65%)	0.1 (<0.01-0.56)	12 (63.16%)	0.01 (<0.01-0.22)	30 (73.17%)	0.15 (<0.01-1.06)	0.062
*Gemella*	18 (56.25%)	0.07 (<0.01-0.31)	14 (35%)	<0.01 (<0.01-0.06)	15 (37.5%)	<0.01 (<0.01-0.22)	15 (37.5%)	<0.01 (<0.01-0.61)	13 (32.5%)	<0.01 (<0.01-0.07)	13 (30.95%)	<0.01 (<0.01-0.1)	24 (63.16%)	0.26 (<0.01-1.22)	18 (45%)	<0.01 (<0.01-0.42)	15 (75%)	0.69 (<0.01-1.55)	12 (63.16%)	0.13 (<0.01-0.9)	28 (68.29%)	0.15 (<0.01-0.68)	<0.001
**Proteobacteria**	32 (100%)	33.57 (27.59-51.89)	40 (100%)	8.49 (3.53-16.01)	40 (100%)	14.35 (10.5-26.58)	40 (100%)	17.66 (11.49-26.26)	40 (100%)	29.81 (8.48-47.97)	42 (100%)	15.25 (8.85-24.36)	38 (100%)	10.62 (3.99-22.95)	40 (100%)	11.5 (4.71-20.89)	20 (100%)	6.74 (2.67-21.41)	19 (100%)	6.9 (2.79-62.38)	41 (100%)	8.25 (3.31-14.05)	<0.001
*Rhodanobacter*	9 (28.12%)	<0.01 (<0.01-0.05)	35 (87.5%)	0.47 (0.06-2.2)	37 (92.5%)	3.38 (1.08-6.81)	31 (77.5%)	3.04 (0.41-5.52)	32 (80%)	0.77 (0.11-6.6)	31 (73.81%)	2.63 (0.28-6.21)	35 (92.11%)	2.55 (0.71-4.43)	27 (67.5%)	0.34 (<0.01-1.49)	11 (55%)	<0.01 (<0.01-0.2)	7 (36.84%)	<0.01 (<0.01-0.05)	30 (73.17%)	0.32 (<0.01-1.66)	<0.001
*Rhizobium*	31 (96.88%)	18.28 (11.16-29.48)	5 (12.5%)	<0.01 (<0.01-<0.01)	4 (10%)	<0.01 (<0.01-<0.01)	2 (5%)	<0.01 (<0.01-<0.01)	10 (25%)	<0.01 (<0.01-0.01)	3 (7.14%)	<0.01 (<0.01-<0.01)	6 (15.79%)	<0.01 (<0.01-<0.01)	4 (10%)	<0.01 (<0.01-<0.01)	2 (10%)	<0.01 (<0.01-<0.01)	7 (36.84%)	<0.01 (<0.01-0.05)	2 (4.88%)	<0.01 (<0.01-<0.01)	<0.001
*Stenotrophomonas*	22 (68.75%)	0.11 (<0.01-0.31)	25 (62.5%)	0.1 (<0.01-0.54)	28 (70%)	0.68 (<0.01-1.89)	24 (60%)	0.45 (<0.01-2.56)	29 (72.5%)	0.27 (<0.01-0.86)	29 (69.05%)	0.4 (<0.01-2.75)	25 (65.79%)	0.08 (<0.01-0.81)	25 (62.5%)	0.31 (<0.01-2.36)	15 (75%)	0.12 (<0.01-0.63)	12 (63.16%)	0.04 (<0.01-0.41)	29 (70.73%)	0.08 (<0.01-0.84)	0.4
*Acinetobacter*	29 (90.62%)	0.95 (0.55-2.91)	25 (62.5%)	0.17 (<0.01-1.12)	30 (75%)	1.31 (0.03-2.39)	27 (67.5%)	0.63 (<0.01-2.48)	29 (72.5%)	0.26 (<0.01-1.25)	21 (50%)	0.03 (<0.01-1.7)	28 (73.68%)	0.82 (0.02-3.94)	24 (60%)	0.14 (<0.01-0.99)	14 (70%)	0.17 (<0.01-0.7)	11 (57.89%)	0.08 (<0.01-1.08)	32 (78.05%)	0.26 (0.01-0.8)	0.004
*Klebsiella*	1 (3.12%)	<0.01 (<0.01-<0.01)	13 (32.5%)	<0.01 (<0.01-0.05)	5 (12.5%)	<0.01 (<0.01-<0.01)	6 (15%)	<0.01 (<0.01-<0.01)	25 (62.5%)	0.64 (<0.01-7.67)	15 (35.71%)	<0.01 (<0.01-0.16)	7 (18.42%)	<0.01 (<0.01-<0.01)	2 (5%)	<0.01 (<0.01-<0.01)	1 (5%)	<0.01 (<0.01-<0.01)	3 (15.79%)	<0.01 (<0.01-<0.01)	1 (2.44%)	<0.01 (<0.01-<0.01)	<0.001
*Achromobacter*	18 (56.25%)	0.62 (<0.01-14.63)	24 (60%)	0.04 (<0.01-0.24)	29 (72.5%)	0.3 (<0.01-1.06)	22 (55%)	0.08 (<0.01-1.22)	22 (55%)	0.02 (<0.01-0.31)	28 (66.67%)	0.13 (<0.01-0.74)	22 (57.89%)	0.07 (<0.01-0.98)	35 (87.5%)	0.45 (0.1-1.39)	13 (65%)	0.09 (<0.01-0.55)	14 (73.68%)	0.08 (<0.01-0.26)	25 (60.98%)	0.07 (<0.01-0.27)	0.005
*Pseudomonas*	15 (46.88%)	<0.01 (<0.01-0.17)	23 (57.5%)	0.09 (<0.01-0.41)	18 (45%)	<0.01 (<0.01-0.72)	23 (57.5%)	0.18 (<0.01-0.68)	26 (65%)	0.08 (<0.01-0.31)	14 (33.33%)	<0.01 (<0.01-0.09)	24 (63.16%)	0.07 (<0.01-0.43)	29 (72.5%)	0.21 (<0.01-0.43)	14 (70%)	0.32 (<0.01-1.04)	15 (78.95%)	0.27 (0.05-1.77)	31 (75.61%)	0.18 (<0.01-0.55)	0.005
**Actinobacteriota**	32 (100%)	16.77 (11.82-28.57)	39 (97.5%)	7.28 (0.58-17.16)	40 (100%)	10.79 (7.5-16.33)	40 (100%)	12.81 (6.21-19.35)	35 (87.5%)	9.37 (1.31-15)	42 (100%)	7.75 (4.95-13.1)	38 (100%)	8.07 (5.04-12.45)	40 (100%)	8.46 (5.72-13.89)	20 (100%)	8.72 (5.78-12.69)	19 (100%)	3.62 (1.45-7.7)	41 (100%)	12.5 (9.21-19.75)	<0.001
*Corynebacterium*	30 (93.75%)	5.04 (2.76-13.33)	28 (70%)	0.63 (<0.01-2.34)	32 (80%)	1.02 (0.31-3.44)	33 (82.5%)	1.12 (0.44-2.49)	30 (75%)	0.78 (0.02-1.49)	35 (83.33%)	2.05 (0.4-3.23)	34 (89.47%)	1.94 (0.55-3.12)	32 (80%)	0.74 (0.09-2.76)	20 (100%)	0.82 (0.44-2.05)	15 (78.95%)	0.3 (0.05-2.53)	37 (90.24%)	1.81 (0.5-4.07)	<0.001
*Cutibacterium*	28 (87.5%)	0.24 (0.11-0.51)	35 (87.5%)	0.36 (0.07-0.73)	37 (92.5%)	1.37 (0.42-2.11)	39 (97.5%)	1.24 (0.54-2.3)	29 (72.5%)	0.57 (<0.01-1.47)	32 (76.19%)	0.37 (0.03-1.24)	36 (94.74%)	0.67 (0.19-1.97)	36 (90%)	0.7 (0.16-2.03)	19 (95%)	1.87 (0.76-3.76)	18 (94.74%)	0.22 (0.08-1.26)	41 (100%)	2.74 (1.14-4.77)	<0.001
*Rothia*	17 (53.12%)	0.04 (<0.01-0.22)	25 (62.5%)	0.17 (<0.01-1.15)	19 (47.5%)	<0.01 (<0.01-0.6)	23 (57.5%)	0.22 (<0.01-1.24)	16 (40%)	<0.01 (<0.01-0.26)	23 (54.76%)	0.3 (<0.01-1.36)	28 (73.68%)	0.82 (<0.01-2.41)	24 (60%)	0.54 (<0.01-2.57)	13 (65%)	0.21 (<0.01-1.27)	10 (52.63%)	0.06 (<0.01-2.05)	27 (65.85%)	0.26 (<0.01-3.53)	0.01
*Bifidobacterium*	30 (93.75%)	1.62 (0.87-2.77)	29 (72.5%)	0.36 (<0.01-1.6)	28 (70%)	0.32 (<0.01-0.95)	25 (62.5%)	0.41 (<0.01-1.44)	29 (72.5%)	0.71 (<0.01-1.69)	30 (71.43%)	0.52 (<0.01-1.22)	25 (65.79%)	0.15 (<0.01-0.49)	29 (72.5%)	0.42 (<0.01-1.36)	15 (75%)	0.54 (0.03-1.36)	12 (63.16%)	0.1 (<0.01-0.42)	29 (70.73%)	0.13 (<0.01-0.37)	<0.001
*Kocuria*	26 (81.25%)	1.28 (0.42-3)	21 (52.5%)	0.03 (<0.01-0.82)	28 (70%)	0.57 (<0.01-1.86)	28 (70%)	0.68 (<0.01-2.24)	22 (55%)	0.09 (<0.01-0.68)	22 (52.38%)	0.25 (<0.01-0.95)	16 (42.11%)	<0.01 (<0.01-0.2)	14 (35%)	<0.01 (<0.01-0.5)	6 (30%)	<0.01 (<0.01-0.36)	2 (10.53%)	<0.01 (<0.01-<0.01)	12 (29.27%)	<0.01 (<0.01-0.14)	<0.001
**Patescibacteria**	16 (50%)	0.02 (<0.01-0.22)	28 (70%)	0.75 (<0.01-5.89)	28 (70%)	0.68 (<0.01-1.63)	40 (100%)	1.37 (<0.01-4.09)	29 (72.5%)	1.35 (<0.01-5.47)	28 (66.67%)	0.25 (<0.01-1.14)	29 (76.32%)	0.55 (0.06-1.57)	38 (95%)	3.21 (1.17-7.57)	19 (95%)	1.97 (0.24-4.31)	14 (73.68%)	0.14 (0.01-1.38)	36 (87.8%)	1.26 (0.37-2.71)	<0.001
Minor_phyla	31 (96.88%)	2.49 (1.55-4.07)	33 (82.5%)	1.53 (0.03-6.13)	39 (97.5%)	4.58 (1.89-6.81)	29 (72.5%)	4.84 (1.86-7.85)	32 (80%)	1.31 (0.03-4.11)	36 (85.71%)	1.85 (0.64-4.91)	38 (100%)	1.9 (0.59-4.78)	39 (97.5%)	4.99 (0.93-6.7)	19 (95%)	1.99 (0.28-4.84)	18 (94.74%)	1.41 (0.26-5.94)	41 (100%)	2.65 (1.2-5.84)	0.003
Minor_genera	32 (100%)	16.89 (11.62-23.67)	36 (90%)	9.93 (2.86-19.82)	40 (100%)	17.28 (9.17-31.39)	40 (100%)	21.36 (12.66-28.95)	38 (95%)	15.51 (3.54-23.46)	42 (100%)	12.57 (8.07-19.24)	38 (100%)	9.16 (3.71-17.33)	40 (100%)	14.46 (5.47-21.38)	20 (100%)	11.31 (3.68-21.06)	18 (94.74%)	8.28 (2.9-15.12)	41 (100%)	13.83 (7.79-22.19)	0.001
Unclassified_phyla	18 (56.25%)	0.01 (<0.01-0.12)	31 (77.5%)	0.39 (0.01-2.01)	40 (100%)	1.46 (0.76-3.29)	40 (100%)	2.09 (0.65-6.13)	32 (80%)	0.54 (0.03-3.65)	42 (100%)	3.04 (1.08-5.12)	38 (100%)	1 (0.4-3.34)	40 (100%)	2.2 (0.69-8.7)	20 (100%)	4.74 (2.56-9.72)	18 (94.74%)	1.21 (0.12-3.77)	41 (100%)	8.44 (4.3-14.33)	<0.001
Unclassified_genera	32 (100%)	3.11 (1.58-4.39)	38 (95%)	5.26 (1.11-16.07)	40 (100%)	8.33 (5.19-11.97)	40 (100%)	10.16 (7.82-14.23)	35 (87.5%)	13.27 (0.29-24.43)	42 (100%)	5.61 (2.98-10.7)	38 (100%)	3.88 (1.52-9.35)	40 (100%)	12.89 (3.42-23.39)	20 (100%)	11.72 (4.08-21.29)	19 (100%)	6.3 (2.52-11.63)	41 (100%)	12.18 (6.07-19.29)	<0.001

**^#^**n (%): number of samples in which the phylum/genus was detected (relative frequency of detection). ^†^Kruskal-Wallis rank tests with Bonferroni correction.

There was an effect of continent on the phyla Proteobacteria and Patescibacteria. More specifically, the abundance of Proteobacteria was higher in African cohorts than the other cohorts (p < 0.001) while that of Patescibacteria was higher in the European samples than in those from the two other continents (p < 0.001). At the genus level, American and European cohorts seemed to be more similar to each other than to the African ones (**Table 2**). African cohorts were characterized by a lower *Streptococcus* abundance (p < 0.001) and a higher *Lactobacillus* abundance (p < 0.001) (**Tables 2** and **3**). The relative abundances of the genera *Rhizobium* and *Corynebacterium* in ETR samples was statistically higher than in any other cohort (p < 0.001) while the contrary was observed for the genus *Rhodanobacter* (p < 0.001) (**Tables 1** and **3**). When European and American cohorts were compared, the relative abundance of the phylum Actinobacteriota was found to be significantly higher in USW cohort than in PE, SP and USC cohorts (p < 0.001), while the relative abundance of the phylum Patescibacteria was higher in SP cohort than in the PE, USW and USC cohorts (p < 0.001) (**Table 4**). At the genus level, the relative abundance of the genus *Streptococcus* was significantly higher in the samples from the PE cohort than in those from the USC, SW and USW cohorts (p < 0.001) (**Table 4**).

**Table 2:** Relative frequencies, medians and interquartile range (IQR) of the most abundant bacterial phyla (bold) and genera detected in milk samples from women in different continents.

Phylum/Genus	AFR		AM		EU		p- value^†^
	n (%)^#^	Median (IQR)	n (%)	Median (IQR)	n (%)	Median (IQR)	
**Firmicutes**	234 (100%)	52.26 (33.5-72.45)	98 (100%)	67.56 (49.7-81.27)	60 (100%)	59.42 (42.99-80.31)	<0.001
*Staphylococcus*	232 (99.15%)	17.89 (7.1-36.2)	96 (97.96%)	20.8 (9.97-35.87)	60 (100%)	22.68 (9.36-38.95)	0.450
*Streptococcus*	217 (92.74%)	8.35 (2.25-19.86)	98 (100%)	21.72 (7.64-43.59)	60 (100%)	12.55 (6.49-35.52)	<0.001
*Lactobacillus*	167 (71.37%)	0.79 (<0.01-3.25)	65 (66.33%)	0.18 (<0.01-0.79)	35 (58.33%)	0.07 (<0.01-0.6)	<0.001
*Veillonella*	114 (48.72%)	<0.01 (<0.01-1.19)	61 (62.24%)	0.16 (<0.01-1.87)	32 (53.33%)	0.02 (<0.01-0.64)	0.089
*Anaerococcus*	130 (55.56%)	0.13 (<0.01-1.17)	68 (69.39%)	0.16 (<0.01-0.89)	31 (51.67%)	0.02 (<0.01-0.69)	0.310
*Gemella*	88 (37.61%)	<0.01 (<0.01-0.2)	64 (65.31%)	0.17 (<0.01-0.93)	33 (55%)	0.03 (<0.01-0.89)	<0.001
**Proteobacteria**	234 (100%)	16.82 (8.65-32.95)	98 (100%)	8.36 (3.32-19.19)	60 (100%)	9.93 (3.74-21.04)	<0.001
*Rhodanobacter*	175 (74.79%)	1.27 (<0.01-5.11)	72 (73.47%)	0.5 (<0.01-2.42)	38 (63.33%)	0.19 (<0.01-1.32)	0.001
*Rhizobium*	55 (23.5%)	<0.01 (<0.01-<0.01)	15 (15.31%)	<0.01 (<0.01-<0.01)	6 (10%)	<0.01 (<0.01-<0.01)	0.012
*Stenotrophomonas*	157 (67.09%)	0.25 (<0.01-1.21)	66 (67.35%)	0.07 (<0.01-0.81)	40 (66.67%)	0.15 (<0.01-1.81)	0.310
*Acinetobacter*	161 (68.8%)	0.51 (<0.01-2.09)	71 (72.45%)	0.3 (<0.01-1.98)	38 (63.33%)	0.15 (<0.01-0.99)	0.170
*Klebsiella*	65 (27.78%)	<0.01 (<0.01-0.05)	11 (11.22%)	<0.01 (<0.01-<0.01)	3 (5%)	<0.01 (<0.01-<0.01)	<0.001
*Achromobacter*	143 (61.11%)	0.07 (<0.01-0.85)	61 (62.24%)	0.07 (<0.01-0.31)	48 (80%)	0.31 (0.03-1.3)	0.017
*Pseudomonas*	119 (50.85%)	0.02 (<0.01-0.4)	70 (71.43%)	0.13 (<0.01-0.56)	43 (71.67%)	0.23 (<0.01-0.78)	0.002
**Actinobacteriota**	228 (97%)	10.5 (5.21-17.74)	98 (100%)	9.21 (5.04-14.75)	60 (100%)	8.46 (5.78-13.89)	0.370
*Corynebacterium*	188 (80.34%)	1.29 (0.21-3.58)	86 (87.76%)	1.69 (0.32-3.46)	52 (86.67%)	0.82 (0.25-2.76)	0.270
*Cutibacterium*	200 (85.47%)	0.56 (0.12-1.61)	95 (96.94%)	1.23 (0.22-2.75)	55 (91.67%)	0.87 (0.25-2.7)	<0.001
*Rothia*	123 (52.56%)	0.08 (<0.01-0.79)	65 (66.33%)	0.41 (<0.01-2.56)	37 (61.67%)	0.37 (<0.01-2.34)	<0.001
*Bifidobacterium*	171 (73.08%)	0.59 (<0.01-1.61)	66 (67.35%)	0.13 (<0.01-0.44)	44 (73.33%)	0.47 (<0.01-1.36)	<0.001
*Kocuria*	147 (62.82%)	0.46 (<0.01-1.5)	30 (30.61%)	<0.01 (<0.01-0.14)	20 (33.33%)	<0.01 (<0.01-0.5)	<0.001
**Patescibacteria**	158 (68%)	0.51 (<0.01-2.38)	79 (81%)	0.73 (0.07-2.07)	57 (95%)	2.44 (0.69-5.87)	<0.001
**Minor_phyla**	211 (90%)	2.67 (0.8-6.08)	97 (99%)	2.21 (0.77-5.54)	58 (97%)	2.8 (0.59-6.36)	0.680
Minor_genera	228 (97.44%)	15.08 (8.34-25.41)	97 (98.98%)	10.65 (4.73-18.84)	60 (100%)	12.2 (5.18-21.38)	0.025
**Unclassified_phyla**	203 (87%)	1.03 (0.12-3.61)	97 (99%)	3.22 (0.59-8.42)	60 (100%)	3.66 (0.76-8.7)	<0.001
Unclassified_genera	227 (97.01%)	7.38 (2.88-13.82)	98 (100%)	8.26 (3.11-13.89)	60 (100%)	12.82 (3.96-22.24)	0.030

**^#^**n (%): number of samples in which the phylum/genus was detected (relative frequency of detection).

^†^Kruskal-Wallis rank tests with Bonferroni correction.

**Table 3:** Relative frequencies, medians and interquartile range (IQR) of the most abundant bacterial phyla (bold) and genera detected in milk samples from African cohorts.

Phylum/Genus	ETR		ETU		GBR		GBU		GN		KE		p-value^†^
	n (%)^#^	Median (IQR)	n (%)	Median (IQR)	n (%)	Median (IQR)	n (%)	Median (IQR)	n (%)	Median (IQR)	n (%)	Median (IQR)	
**Firmicutes**	32 (100%)	34.13 (21.56-51.3)	40 (100%)	64.65 (36.57-82.26)	40 (100%)	63.34 (43.84-75.13)	39 (97.5%)	53.67 (44.13-62.98)	40 (100%)	37.29 (24.44-71.19)	42 (100%)	69.21 (48.17-74.24)	<0.001
*Staphylococcus*	32 (100%)	9.67 (5.42-16.39)	40 (100%)	29.92 (8.78-48.42)	39 (97.5%)	14.81 (6.96-35.69)	40 (100%)	22.98 (16.06-34.3)	39 (97.5%)	9.59 (3.86-24.52)	42 (100%)	28.49 (13.47-52.45)	<0.001
*Streptococcus*	30 (93.75%)	7.3 (3.28-13.29)	35 (87.5%)	5.91 (0.15-16.78)	39 (97.5%)	9.33 (3.59-32.28)	37 (92.5%)	10.25 (4.75-21.88)	36 (90%)	2.49 (0.08-8.88)	40 (95.24%)	15.43 (5.38-29.94)	<0.001
*Lactobacillus*	26 (81.25%)	1.03 (0.25-2.93)	24 (60%)	0.13 (<0.01-0.51)	31 (77.5%)	1.57 (0.06-3.68)	28 (70%)	1.7 (<0.01-5.58)	30 (75%)	1.24 (0.01-5.01)	28 (66.67%)	0.79 (<0.01-2.77)	0.003
*Veillonella*	24 (75%)	0.75 (0.04-4.11)	18 (45%)	<0.01 (<0.01-0.28)	24 (60%)	0.3 (<0.01-1.52)	18 (45%)	<0.01 (<0.01-2.31)	12 (30%)	<0.01 (<0.01-0.13)	18 (42.86%)	<0.01 (<0.01-1.18)	<0.001
*Anaerococcus*	23 (71.88%)	1.21 (<0.01-3.15)	21 (52.5%)	0.05 (<0.01-0.38)	24 (60%)	0.14 (<0.01-1.21)	20 (50%)	0.03 (<0.01-1.38)	24 (60%)	0.2 (<0.01-0.53)	18 (42.86%)	<0.01 (<0.01-0.92)	0.025
*Gemella*	18 (56.25%)	0.07 (<0.01-0.31)	14 (35%)	<0.01 (<0.01-0.06)	15 (37.5%)	<0.01 (<0.01-0.22)	15 (37.5%)	<0.01 (<0.01-0.61)	13 (32.5%)	<0.01 (<0.01-0.07)	13 (30.95%)	<0.01 (<0.01-0.1)	0.010
**Proteobacteria**	32 (100%)	33.57 (27.59-51.89)	40 (100%)	8.49 (3.53-16.01)	40 (100%)	14.35 (10.5-26.58)	40 (100%)	17.66 (11.49-26.26)	40 (100%)	29.81 (8.48-47.97)	42 (100%)	15.25 (8.85-24.36)	<0.001
*Rhodanobacter*	9 (28.12%)	<0.01 (<0.01-0.05)	35 (87.5%)	0.47 (0.06-2.2)	37 (92.5%)	3.38 (1.08-6.81)	31 (77.5%)	3.04 (0.41-5.52)	32 (80%)	0.77 (0.11-6.6)	31 (73.81%)	2.63 (0.28-6.21)	<0.001
*Rhizobium*	31 (96.88%)	18.28 (11.16-29.48)	5 (12.5%)	<0.01 (<0.01-<0.01)	4 (10%)	<0.01 (<0.01-<0.01)	2 (5%)	<0.01 (<0.01-<0.01)	10 (25%)	<0.01 (<0.01-0.01)	3 (7.14%)	<0.01 (<0.01-<0.01)	<0.001
*Stenotrophomonas*	22 (68.75%)	0.11 (<0.01-0.31)	25 (62.5%)	0.1 (<0.01-0.54)	28 (70%)	0.68 (<0.01-1.89)	24 (60%)	0.45 (<0.01-2.56)	29 (72.5%)	0.27 (<0.01-0.86)	29 (69.05%)	0.4 (<0.01-2.75)	<0.001
*Acinetobacter*	29 (90.62%)	0.95 (0.55-2.91)	25 (62.5%)	0.17 (<0.01-1.12)	30 (75%)	1.31 (0.03-2.39)	27 (67.5%)	0.63 (<0.01-2.48)	29 (72.5%)	0.26 (<0.01-1.25)	21 (50%)	0.03 (<0.01-1.7)	<0.001
*Klebsiella*	1 (3.12%)	<0.01 (<0.01-<0.01)	13 (32.5%)	<0.01 (<0.01-0.05)	5 (12.5%)	<0.01 (<0.01-<0.01)	6 (15%)	<0.01 (<0.01-<0.01)	25 (62.5%)	0.64 (<0.01-7.67)	15 (35.71%)	<0.01 (<0.01-0.16)	<0.001
*Achromobacter*	18 (56.25%)	0.62 (<0.01-14.63)	24 (60%)	0.04 (<0.01-0.24)	29 (72.5%)	0.3 (<0.01-1.06)	22 (55%)	0.08 (<0.01-1.22)	22 (55%)	0.02 (<0.01-0.31)	28 (66.67%)	0.13 (<0.01-0.74)	<0.001
*Delftia*	23(71.88%)	0.37 (<0.01-2.37)	22(55.00%)	0.01 (<0.01-0.15)	22(55.00%)	0.28 (<0.01-1.18)	21(52.50%)	0.02 (<0.01-1.06)	23(57.50%)	0.01 (<0.01-0.39)	33(78.57%)	0.43 (0.07-1.31)	0.005
**Actinobacteriota**	32 (100%)	16.77 (11.82-28.57)	39 (97.5%)	7.28 (0.58-17.16)	40 (100%)	10.79 (7.5-16.33)	40 (100%)	12.81 (6.21-19.35)	35 (87.5%)	9.37 (1.31-15)	42 (100%)	7.75 (4.95-13.1)	<0.001
*Corynebacterium*	30 (93.75%)	5.04 (2.76-13.33)	28 (70%)	0.63 (<0.01-2.34)	32 (80%)	1.02 (0.31-3.44)	33 (82.5%)	1.12 (0.44-2.49)	30 (75%)	0.78 (0.02-1.49)	35 (83.33%)	2.05 (0.4-3.23)	<0.001
*Cutibacterium*	28 (87.5%)	0.24 (0.11-0.51)	35 (87.5%)	0.36 (0.07-0.73)	37 (92.5%)	1.37 (0.42-2.11)	39 (97.5%)	1.24 (0.54-2.3)	29 (72.5%)	0.57 (<0.01-1.47)	32 (76.19%)	0.37 (0.03-1.24)	<0.001
*Rothia*	17 (53.12%)	0.04 (<0.01-0.22)	25 (62.5%)	0.17 (<0.01-1.15)	19 (47.5%)	<0.01 (<0.01-0.6)	23 (57.5%)	0.22 (<0.01-1.24)	16 (40%)	<0.01 (<0.01-0.26)	23 (54.76%)	0.3 (<0.01-1.36)	0.18
*Bifidobacterium*	30 (93.75%)	1.62 (0.87-2.77)	29 (72.5%)	0.36 (<0.01-1.6)	28 (70%)	0.32 (<0.01-0.95)	25 (62.5%)	0.41 (<0.01-1.44)	29 (72.5%)	0.71 (<0.01-1.69)	30 (71.43%)	0.52 (<0.01-1.22)	<0.001
*Kocuria*	26 (81.25%)	1.28 (0.42-3)	21 (52.5%)	0.03 (<0.01-0.82)	28 (70%)	0.57 (<0.01-1.86)	28 (70%)	0.68 (<0.01-2.24)	22 (55%)	0.09 (<0.01-0.68)	22 (52.38%)	0.25 (<0.01-0.95)	<0.001
**Patescibacteria**	16 (50%)	0.02 (<0.01-0.22)	28 (70%)	0.75 (<0.01-5.89)	28 (70%)	0.68 (<0.01-1.63)	40 (100%)	1.37 (<0.01-4.09)	29 (72.5%)	1.35 (<0.01-5.47)	28 (66.67%)	0.25 (<0.01-1.14)	<0.001
Minor_Phyla	31 (96.88%)	2.49 (1.55-4.07)	33 (82.5%)	1.53 (0.03-6.13)	39 (97.5%)	4.58 (1.89-6.81)	29 (72.5%)	4.84 (1.86-7.85)	32 (80%)	1.31 (0.03-4.11)	36 (85.71%)	1.85 (0.64-4.91)	<0.001
Minor_genera	32 (100%)	16.89 (11.62-23.67)	36 (90%)	9.93 (2.86-19.82)	40 (100%)	17.28 (9.17-31.39)	40 (100%)	21.36 (12.66-28.95)	38 (95%)	15.51 (3.54-23.46)	42 (100%)	12.57 (8.07-19.24)	0.002
Unclassified_phyla	18 (56.25%)	0.01 (<0.01-0.12)	31 (77.5%)	0.39 (0.01-2.01)	40 (100%)	1.46 (0.76-3.29)	40 (100%)	2.09 (0.65-6.13)	32 (80%)	0.54 (0.03-3.65)	42 (100%)	3.04 (1.08-5.12)	<0.001
Unclassified_genera	32 (100%)	3.11 (1.58-4.39)	38 (95%)	5.26 (1.11-16.07)	40 (100%)	8.33 (5.19-11.97)	40 (100%)	10.16 (7.82-14.23)	35 (87.5%)	13.27 (0.29-24.43)	42 (100%)	5.61 (2.98-10.7)	<0.001

**^#^**n (%): number of samples in which the phylum/genus was detected (relative frequency of detection).

^†^Kruskal-Wallis rank tests with Bonferroni correction.

**Table 4:** Relative frequencies, medians and interquartile range (IQR) of the most abundant bacterial phyla (bold) and genera detected in milk samples from European and American cohorts.

Phylum/Genus	SP		SW		PE		USC		USW		P-value^†^
	n (%)	n (%)	n (%)	Median (IQR)	n (%)	Median (IQR)	n (%)	Median (IQR)	n (%)	Median (IQR)	
**Firmicutes**	40 (100%)	52.34 (41.38-80.31)	20 (100%)	63.1 (52.14-80.37)	38 (100%)	73.74 (53.5-85.68)	19 (100%)	72.95 (18.73-83.59)	41 (100%)	60.58 (48.72-70.05)	0.074
*Staphylococcus*	40 (100%)	14.78 (7.51-33.91)	20 (100%)	26.32 (20.33-49.74)	36 (94.74%)	18.12 (8.19-31.03)	19 (100%)	10.49 (4.69-51.83)	41 (100%)	25.26 (12.76-45.55)	0.12
*Streptococcus*	40 (100%)	12.55 (5.39-52.6)	20 (100%)	11.97 (7.84-28.44)	38 (100%)	37.8 (22.74-57.26)	19 (100%)	11.38 (1.82-31.85)	41 (100%)	15.57 (6.59-28.15)	<0.001
*Lactobacillus*	18 (45%)	<0.01 (<0.01-0.41)	17 (85%)	0.3 (0.05-1.49)	23 (60.53%)	0.08 (<0.01-1.01)	11 (57.89%)	0.02 (<0.01-0.25)	31 (75.61%)	0.44 (0.01-1.28)	0.016
*Veillonella*	18 (45%)	<0.01 (<0.01-0.59)	14 (70%)	0.15 (<0.01-0.72)	27 (71.05%)	1.43 (<0.01-4.08)	12 (63.16%)	0.06 (<0.01-1.54)	22 (53.66%)	0.01 (<0.01-0.6)	0.006
*Anaerococcus*	18 (45%)	<0.01 (<0.01-0.78)	13 (65%)	0.1 (<0.01-0.56)	26 (68.42%)	0.3 (<0.01-0.95)	12 (63.16%)	0.01 (<0.01-0.22)	30 (73.17%)	0.15 (<0.01-1.06)	0.33
*Gemella*	18 (45%)	<0.01 (<0.01-0.42)	15 (75%)	0.69 (<0.01-1.55)	24 (63.16%)	0.26 (<0.01-1.22)	12 (63.16%)	0.13 (<0.01-0.9)	28 (68.29%)	0.15 (<0.01-0.68)	0.18
**Proteobacteria**	40 (100%)	11.5 (4.71-20.89)	20 (100%)	6.74 (2.67-21.41)	38 (100%)	10.62 (3.99-22.95)	19 (100%)	6.9 (2.79-62.38)	41 (100%)	8.25 (3.31-14.05)	0.81
*Rhodanobacter*	27 (67.5%)	0.34 (<0.01-1.49)	11 (55%)	<0.01 (<0.01-0.2)	35 (92.11%)	2.55 (0.71-4.43)	7 (36.84%)	<0.01 (<0.01-0.05)	30 (73.17%)	0.32 (<0.01-1.66)	<0.001
*Rhizobium*	4 (10%)	<0.01 (<0.01-<0.01)	2 (10%)	<0.01 (<0.01-<0.01)	6 (15.79%)	<0.01 (<0.01-<0.01)	7 (36.84%)	<0.01 (<0.01-0.05)	2 (4.88%)	<0.01 (<0.01-<0.01)	<0.001
*Stenotrophomonas*	25 (62.5%)	0.31 (<0.01-2.36)	15 (75%)	0.12 (<0.01-0.63)	25 (65.79%)	0.08 (<0.01-0.81)	12 (63.16%)	0.04 (<0.01-0.41)	29 (70.73%)	0.08 (<0.01-0.84)	0.87
*Acinetobacter*	24 (60%)	0.14 (<0.01-0.99)	14 (70%)	0.17 (<0.01-0.7)	28 (73.68%)	0.82 (0.02-3.94)	11 (57.89%)	0.08 (<0.01-1.08)	32 (78.05%)	0.26 (0.01-0.8)	0.28
*Delftia*	34 (85%)	<0.01 (<0.01-<0.01)	15 (75%)	0.1 (0.01-0.41)	17 (44.74%)	<0.01 (<0.01-0.89)	10 (52.63%)	0.01 (<0.01-0.14)	25 (60.98%)	0.09 (<0.01-0.45)	0.002
*Achromobacter*	35 (87.5%)	0.45 (0.1-1.39)	13 (65%)	0.09 (<0.01-0.55)	22 (57.89%)	0.07 (<0.01-0.98)	14 (73.68%)	0.08 (<0.01-0.26)	25 (60.98%)	0.07 (<0.01-0.27)	0.007
*Pseudomonas*	29 (72.5%)	0.21 (<0.01-0.43)	14 (70%)	0.32 (<0.01-1.04)	24 (63.16%)	0.07 (<0.01-0.43)	15 (78.95%)	0.27 (0.05-1.77)	31 (75.61%)	0.18 (<0.01-0.55)	0.36
**Actinobacteriota**	40 (100%)	8.46 (5.72-13.89)	20 (100%)	8.72 (5.78-12.69)	38 (100%)	8.07 (5.04-12.45)	19 (100%)	3.62 (1.45-7.7)	41 (100%)	12.5 (9.21-19.75)	<0.001
*Corynebacterium*	32 (80%)	0.74 (0.09-2.76)	20 (100%)	0.82 (0.44-2.05)	34 (89.47%)	1.94 (0.55-3.12)	15 (78.95%)	0.3 (0.05-2.53)	37 (90.24%)	1.81 (0.5-4.07)	0.1
*Cutibacterium*	36 (90%)	0.7 (0.16-2.03)	19 (95%)	1.87 (0.76-3.76)	36 (94.74%)	0.67 (0.19-1.97)	18 (94.74%)	0.22 (0.08-1.26)	41 (100%)	2.74 (1.14-4.77)	<0.001
*Rothia*	24 (60%)	0.54 (<0.01-2.57)	13 (65%)	0.21 (<0.01-1.27)	28 (73.68%)	0.82 (<0.01-2.41)	10 (52.63%)	0.06 (<0.01-2.05)	27 (65.85%)	0.26 (<0.01-3.53)	0.7
*Bifidobacterium*	29 (72.5%)	0.42 (<0.01-1.36)	15 (75%)	0.54 (0.03-1.36)	25 (65.79%)	0.15 (<0.01-0.49)	12 (63.16%)	0.1 (<0.01-0.42)	29 (70.73%)	0.13 (<0.01-0.37)	0.096
*Kocuria*	14 (35%)	<0.01 (<0.01-0.5)	6 (30%)	<0.01 (<0.01-0.36)	16 (42.11%)	<0.01 (<0.01-0.2)	2 (10.53%)	<0.01 (<0.01-<0.01)	12 (29.27%)	<0.01 (<0.01-0.14)	<0.001
**Patescibacteria**	38 (95%)	3.21 (1.17-7.57)	19 (95%)	1.97 (0.24-4.31)	29 (76.32%)	0.55 (0.06-1.57)	14 (73.68%)	0.14 (0.01-1.38)	36 (87.8%)	1.26 (0.37-2.71)	<0.001
Minor_Phyla	39 (97.5%)	4.99 (0.93-6.7)	19 (95%)	1.99 (0.28-4.84)	38 (100%)	1.9 (0.59-4.78)	18 (94.74%)	1.41 (0.26-5.94)	41 (100%)	2.65 (1.2-5.84)	<0.001
*Flavobacterium*	28 (70%)	14.46 (5.47-21.38)	6 (30%)	<0.01 (<0.01-0.02)	8 (21.05%)	<0.01 (<0.01-<0.01)	6 (31.58%)	<0.01 (<0.01-0.14)	11 (26.83%)	<0.01 (<0.01-0.01)	0.003
Minor_genera	40 (100%)	2.2 (0.69-8.7)	20 (100%)	11.31 (3.68-21.06)	38 (100%)	9.16 (3.71-17.33)	18 (94.74%)	8.28 (2.9-15.12)	41 (100%)	13.83 (7.79-22.19)	0.34
Unclassified_phyla	40 (100%)	12.89 (3.42-23.39)	20 (100%)	4.74 (2.56-9.72)	38 (100%)	1 (0.4-3.34)	18 (94.74%)	1.21 (0.12-3.77)	41 (100%)	8.44 (4.3-14.33)	<0.001
Unclassified_genera	40 (100%)	52.34 (41.38-80.31)	20 (100%)	11.72 (4.08-21.29)	38 (100%)	3.88 (1.52-9.35)	19 (100%)	6.3 (2.52-11.63)	41 (100%)	12.18 (6.07-19.29)	0.001

**^#^**n (%): number of samples in which the phylum/genus was detected (relative frequency of detection).

^†^Kruskal-Wallis rank tests with Bonferroni correction.

### Comparison of the Results Obtained With the Two Strategies (Sequencing of V1-V3 Versus V3-V4 Region) With the Same Database (SILVA 132)

Overall, the comparison of the results obtained by Lackey et al. (2019) targeting the V1-V3 region and those obtained in this work, targeting the V3-V4 region, showed notable differences among the most abundant phyla and genera. However, some of these differences were the result of different nomenclatures used by SILVA 132 (used in Lackey et al., 2019) and SILVA 138 (used in the present study) database versions, as is the case of Actinobacteriota and Actinobacteria; or the genera *Propionibacterium* and *Cutibacterium*, in which the pipeline considers them as different microorganisms.

To avoid this bias and facilitate comparison among both studies, we re-analyzed our sequences on the V3-V4 regions with the same database used by Lackey and colleagues (SILVA 132). For this comparison reads that could not be classified to the genus level were included. Finally, a total of 26,461,984 high quality reads were used to perform this comparison (**Supplementary Table 3**).

**Figure 4:**
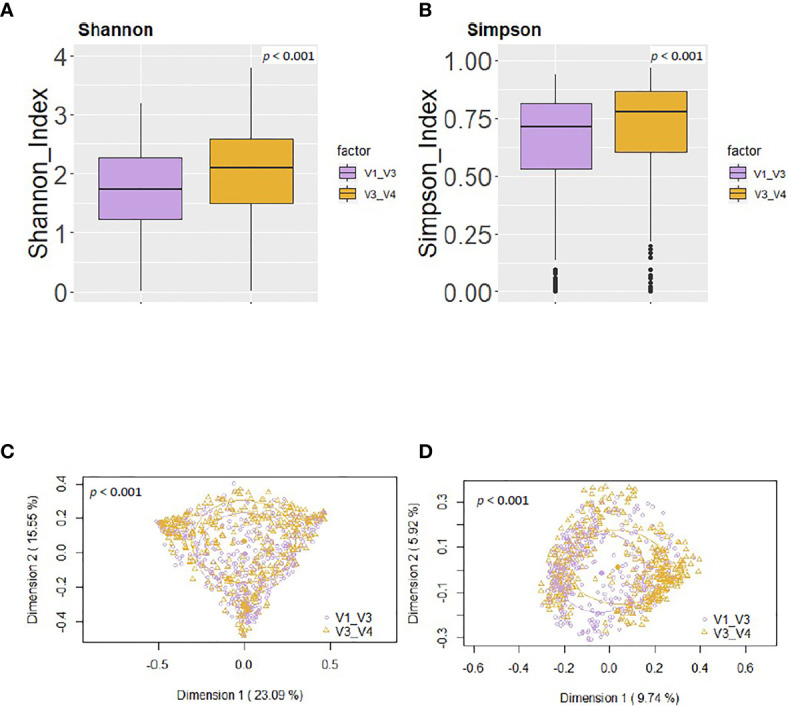
Comparison between the values of alpha and beta diversity obtained after the analysis at the genus level of the same set of milk samples, either with the V3-V4 (this work) or the V1-V3 (Lackey et al., 2019) approach. **(A)** Shannon diversity index; **(B)** Simpson diversity index; **(C)** PCoA plots based on the Bray-Curtis dissimilarity index; **(D)** PCoA plots based on the Jaccard’s coefficient with the SILVA 132 database.

Using the same reference database (SILVA 132) and post-taxonomic bioinformatic analysis pipeline yielded some specific differences in the alpha diversity results. In general, the V3-V4 16S rRNA region study showed a higher alpha-diversity [Shannon and Simpson indices 2.03 (1.46-2.49) and 0.77 (0.59-0.85) respectively] than V1-V3 study [Shannon and Simpson indices 1.74 (1.21-2.28) and 0.71 (0.53-0.81) respectively] (p < 0.001) (**Figures 4A, B**). In addition, PCoA plots based on the Bray-Curtis dissimilarity index (**Figure 4C**) and on the Jaccard’s coefficient (**Figure 4D**) revealed differences in the beta diversity results.

In contrast, both sequencing approaches led to highly concordant results in relation to some individual phyla and genera, allowing similar comparisons across cohorts. For instance, in terms of individual phyla, in both studies Firmicutes, Proteobacteria, and Actinobacteriota collectively represented >90% of those identified (**Table 5**). Besides, both 16S region studies showed that the relative abundance of Firmicutes was lower in ETR than in all cohorts (p < 0.001), that Proteobacteria was relatively more abundant in milk collected in ETR than in all other cohorts (p < 0.001), and that Actinobacteriota was more abundant in ETR, ETU, GBR, and GBU than in GN, USC, SP, and PE (p ≤ 0.001). The higher relative abundance of Bacteroidetes in KE than GN was also detected in both studies (p < 0.001). There was also an effect of cohort on the “other” (Lackey et al., 2019) and “minor_phyla” (this work) category; which relative abundance in GBU was higher than in ETR, ETU, GN, PE, SP, SW, and USC (p ≤ 0.001) according to both strategies.

**Table 5:** Relative frequencies, medians and interquartile range (IQR) of the most abundant bacterial phyla (bold) and genera detected in the milk samples analyzed either with the V3-V4 (this work) or the V1-V3 (Lackey et al., 2019) approach with SILVA 132 database.

Phylum (bold)/Genus	V1-V3		V3-V4		P-value^†^
	n (%)^#^	Median (IQR)	n (%)	Median (IQR)	
Firmicutes	394 (100%)	63.03 (34.37-84.33)	392 (100%)	56.69 (41.15-76.09)	0.23
*Staphylococcus*	389 (98.73%)	12.49 (3.63-33.51)	389 (99.23%)	19.3 (8.03-36.84)	<0.001
*Streptococcus*	387 (98.22%)	15.61 (4.81-41.6)	377 (96.17%)	10.75 (3.62-28.16)	0.001
*Lactobacillus*	185 (46.95%)	<0.01 (<0.01-0.62)	280 (71.43%)	0.46 (<0.01-2.3)	<0.001
*Veillonella*	229 (58.12%)	0.25 (<0.01-1.62)	207 (52.81%)	0.04 (<0.01-1.28)	0.051
*Gemella*	189 (47.97%)	<0.01 (<0.01-0.74)	186 (47.45%)	<0.01 (<0.01-0.61)	0.380
Proteobacteria	394 (100%)	9.18 (3.47-25.32)	392 (100%)	13.84 (6.24-26.73)	0.002
*Acinetobacter*	204 (51.78%)	0.06 (<0.01-0.59)	270 (68.88%)	0.37 (<0.01-1.97)	<0.001
*Rhizobium*	115 (29.19%)	<0.01 (<0.01-0.05)	79 (20.15%)	<0.01 (<0.01-<0.01)	0.004
*Klebsiella*	51 (12.94%)	<0.01 (<0.01-<0.01)	82 (20.92%)	<0.01 (<0.01-<0.01)	0.004
*Rhodanobacter*	3 (0.76%)	<0.01 (<0.01-<0.01)	286 (72.96%)	0.66 (<0.01-3.68)	<0.001
*Stenotrophomonas*	137 (34.77%)	<0.01 (<0.01-0.2)	264 (67.35%)	0.14 (<0.01-1.1)	<0.001
*Achromobacter*	51 (12.94%)	<0.01 (<0.01-<0.01)	255 (65.05%)	0.1 (<0.01-0.76)	<0.001
*Dyella*	272 (69.04%)	0.83 (<0.01-2.57)	2 (0.51%)	<0.01 (<0.01-<0.01)	<0.001
Actinobacteria	381 (96.7%)	13.23 (3.95-25.09)	386 (98.47%)	9.93 (5.39-16.65)	0.003
*Corynebacterium_1*	295 (74.87%)	1.03 (<0.01-5.49)	324 (82.65%)	1.19 (0.27-3.33)	0.88
*Rothia*	256 (64.97%)	0.38 (<0.01-2.32)	224 (57.14%)	0.15 (<0.01-1.29)	<0.001
*Propionibacterium*	307 (77.92%)	0.49 (0.07-2.19)	0 (0%)	<0.01 (<0.01-<0.01)	<0.001
*Kocuria*	175 (44.42%)	<0.01 (<0.01-0.75)	196 (50%)	0.01 (<0.01-1.05)	0.120
*Bifidobacterium*	198 (50.25%)	0.02 (<0.01-0.94)	285 (72.7%)	0.42 (<0.01-1.32)	<0.001
*Cutibacterium*	0 (0%)	<0.01 (<0.01-<0.01)	355 (90.56%)	0.75 (0.18-2.1)	<0.001
Bacteroidetes	334 (84.77%)	0.65 (0.16-1.96)	321 (81.89%)	1.02 (0.12-2.74)	0.049
Minor_phyla	319 (80.96%)	0.4 (0.04-1.32)	350 (89.29%)	2.34 (0.52-5.91)	<0.001
Minor_genera	393 (99.75%)	9.98 (3.69-20.51)	387 (98.72%)	18.86 (8.74-30.12)	<0.001
Unclassified_phyla	283 (71.83%)	0.24 (<0.01-2.03)	360 (91.84%)	1.53 (0.28-5.31)	<0.001
Unclassified_genera	376 (95.43%)	3.33 (1.32-11.6)	383 (97.7%)	4.49 (1.76-10.51)	0.29

**^#^**n (%): number of samples in which the phylum/genus was detected (relative frequency of detection).

^†^Wilconxon rank tests with Bonferroni correction.

At the individual genus analysis, both approaches found a higher relative abundance of *Rhizobium, Achromobacter*, and *Corynebacterium* in milk collected in ETR than all other cohorts. Besides, statistical differences were found in almost all pairwise comparisons (p < 0.05) with ETR V1-V3 region strategy, except for *Corynebacterium* with ETU. Both V1-V3 and V3-V4 approaches showed that African cohorts had the lowest abundance of *Streptococcus* (p < 0.05) while Peruvian milk bacterial communities had the highest relative abundance of this genus as compared to all the other cohorts (p < 0.05) in a pairwise comparison.

In our work, *Lactobacillus* had a higher relative abundance among some African cohorts (particularly in ETR, GBR, GBU and GN) than all other cohorts but these differences were not statistically significant. Interestingly, in agreement with these observations, Lackey et al. (2019) also analyzed the fecal microbiome of the breastfed babies and found a higher relative abundance of *Lactobacillus* in feces of ETR, GBR and GBU than in samples from the PE, SP, SW and US cohorts.

**Figure 5:**
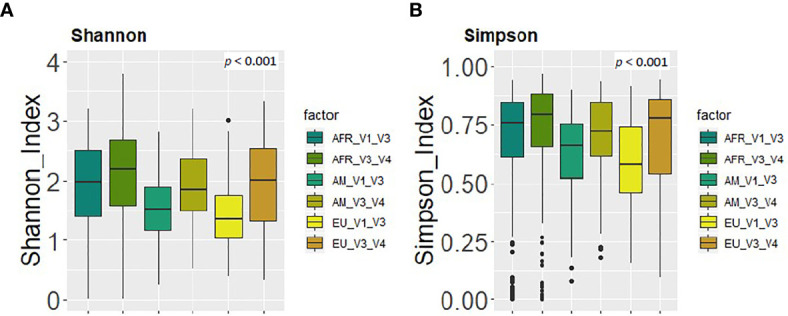
Comparison between the values of alpha diversity obtained after the analysis at the genus level of the same set of milk samples, either with the V3-V4 (this work) or the V1-V3 (Lackey et al., 2019) approach and continent cohorts. **(A)** Shannon diversity index; **(B)** Simpson diversity index.

Finally, in relation to alpha and beta diversity analysis, both approaches found that African samples, and particularly those from ETR, displayed the highest alpha diversity as assessed by the Shannon and Simpson indices (**Figures 5A, B**). Comparison of the mean distances of samples to the centroids using PCoA plots based either on the Bray-Curtis dissimilarity index or on the Jaccard’s coefficient of each cohort, revealed no differences between both approaches (p=0.87 and p=0.37, respectively).

On the other hand, some differences were observed among the results obtained by Lackey et al. (2019) and those obtained in this work (**Table 5**). The relative abundance of the phylum Proteobacteria detected in our work was higher than in Lackey et al. (2019) [13.84 (6.24-26.73) *vs* 9.18 (3.47-25.32), p = 0.002]. On the contrary, the phylum Actinobacteriota was higher in Lackey et al. (2019) than in our work [13.23 (3.95-25.09) *vs* 9.93 (5.39-16.65), p = 0.003] (**Table 5**), whereas the medians of the Shannon or Simpson diversity indices were higher in this work (p < 0.001; Kruskal–Wallis tests with Bonferroni correction) (**Figures 4** and **5**). It is also worth noting that, despite finding comparable microbial community structures with both sequencing approaches, significant differences were found in the detection of the relative abundance of some bacterial genera which can be of great importance for the maternal-infant health, such as *Staphylococcus, Streptococcus, Bifidobacterium* and *Lactobacillus* (**Table 5**).

## Discussion

Findings from this study expand upon the milk microbiota profiling previously reported from the INSPIRE cohort (Lackey et al., 2019), which is the most comprehensive study to date on the topic. In addition, our findings provide, for the first time and across globally diverse populations, evidence of the impact of different DNA processing and sequencing approaches on the microbiota profiles obtained for human milk samples. While comparison of sequencing approaches and DNA isolation procedures has been long studied in the context of its impact on the GI microbiome (Hill et al., 2016; Rintala et al., 2017; Panek et al., 2018), little effort has been devoted to achieving standardization of optimal procedures to tackle the study of the human milk microbiome. However, such standardization is required to enable meaningful comparisons of the datasets generated across different studies of these important microbial communities, especially considering particular intrinsic characteristics which may impose additional challenges for microbiome studies. First, human milk has a relatively low density of bacterial cells (between 10^3^ to 10^5^ cfu mL^-1^), can have a relatively high concentration of immune cells (and thus human DNA), and a high fat concentration that might entrap some bacterial cells. In addition, there exists an important risk of cross contamination of the samples with skin samples during collection or due to exposure to breast pumps; all of these factors can potentially introduce biases in microbiota studies (Boix-Amorós et al., 2016; Moossavi and Azad, 2019). In this context, our hypothesis is that DNA isolation, library generation, sequencing approach and data analysis can significantly impact the human milk microbiota profiles obtained through HTS surveys. For this purpose, the same set of human milk samples previously studied by means of sequencing the 16S rRNA V1-V3 regions reported by Lackey and colleagues (2019) was herein re-extracted and re-sequenced using different methods. For this study, the milk samples were extracted using the QIAamp DNA Stool Mini Kit with additional mechanical bead beating and using an amplicon sequencing approach that targeted a different 16S rRNA variable region (V3-V4) and that achieved higher sequencing depth. For the purposes of comparison, both datasets were downstream processed through identical post-taxonomy bioinformatics pipelines and reads generated in the present study (V3-V4 regions) were taxonomically re-assigned against the same reference database used in Lackey et al. (2019).

Overall, the dataset presented herein confirms the existence of large inter-individual and inter-populations variations in the healthy human milk microbiota across diverse geographical and ethnical populations as previously reported in several studies (Kumar et al., 2016; Vaidya et al., 2017). In agreement to the dataset previously reported by Lackey and colleagues on the same set of samples, this variation was evident between geographically distant but also, to some extent, between neighboring populations. For instance, both sequencing approaches found that the microbial communities present in African cohorts were dissimilar to those found in the European and American cohorts. The African cohorts also displayed higher diversity of microbial taxa, with ETR being the population harboring the most distinctive microbiota fingerprint. It must be highlighted that due to limitations in electricity access at ETR, this set of samples was preserved at room temperature in a preservation solution immediately following collection and thus this data must be interpreted with caution. While the utilization of a different preservation and DNA isolation procedure might have introduced bias in the microbiota profiles detected for this particular cohort which could be partially responsible for the high dissimilarity exhibited by the ETR cohort as compared to the rest of the dataset, at this moment we cannot rule out to what extent these dissimilarities are due to genuinely biological differences. Nonetheless, it is worth remarking that other African cohorts also displayed significantly different beta-diversities as compared to American and European cohorts, particularly in terms of presence/absence of taxa, and that these observations were consistent among both 16S rRNA sequencing datasets. Besides, ETR together with GBU and GN were the cohorts displaying the highest alpha-diversity indices across all the studied cohorts. Overall, these observations agree with the overall reported loss of diversity in human microbiomes from populations with westernized and industrialized lifestyles (Segata, 2015), further suggesting that common genetic, environmental and/or lifestyle factors might have influenced a differential composition in the human milk microbiota in the cohorts under study. In line with “The Hygiene Hypothesis”, these observations likely reflect a broad exposure to a wider array of microorganisms in some African cohorts as opposed to other European or American cohorts where westernized practices such as antibiotic utilization, water sanitation or reduced contact with animals, among others, may have reduced diversity in the human-associated microbial communities and the concomitant increase in non-communicable chronic diseases (Sonnenburg et al., 2016; Vandegrift et al., 2017). Further, even neighboring populations with different lifestyles, such as those represented by rural and urban communities of Ethiopia and rural and urban communities of Gambia, still exhibited significant differences in the diversity and/or structure of their respective milk microbiotas. These results support those reported by other authors when comparing the milk microbiota in urban and rural populations in India and China (Li et al., 2017; Vaidya et al., 2017). These observations reinforce the notion that microbial exposure, environmental factors and lifestyle habits strongly impact the assemblage of the human milk microbiome (Lackey et al., 2019) and, due to the influence of these microbial communities on seeding the infant GI microbiome; such factors might have decisive implications in infant health outcomes (Browne et al., 2019).

It is also worth noting that some African cohorts including ETR, GN and GBU presented taxa that were exclusively associated to their respective cohorts. For instance, GN samples were the only ones in which *Akkermansia* and *Butyricicoccus* were detected, both groups representing bacteria with attributed health promoting effects in models of inflammatory bowel disease (Eeckhaut et al., 2013; Ferrer-Picón et al., 2020), and likely representing candidates to develop prospective next-generation probiotics (O’Toole et al., 2017). The representation in the milk microbiota of other taxa traditionally including commensal microbes with attributed health promoting effects, such as *Lactobacillus* and *Bifidobacterium*, was also higher among African cohorts; and this result was independent on the sequencing approach employed. These observations strengthen recent research trends that defend the necessity to capture and preserve the microbial diversity from globally diverse human populations, as they might include taxa that could help mitigate non-communicable and chronic human diseases highly prevalent in industrialized and urbanized western populations (Dominguez-Bello et al., 2018; Sonnenburg and Sonnenburg, 2019).

The current dataset also supports the existence of a few universal core taxa consisting of dominant bacterial groups such as *Staphylococcus* and *Streptococcus*; as well as some other taxa that, despite being present in over 70% of the analyzed samples, exhibited minor relative abundances such as *Cutibacterium, Corynebacterium, Rhodanobacter* and *Bifidobacterium.* Some population-specific core taxa were also identified for some cohorts, in accordance with the results reported by Lackey et al., although population-specific core taxa appeared different depending on the sequencing approach. Moreover, the ETR core included *Rhizobium, Bifidobacterium* and *Acinetobacter*, the last two genera not present in the ETR core microbiota based on V1-V3 results. The prevalence of these bacterial groups likely drives the beta-diversity differentiation of this particular cohort from the other populations analyzed.

In conclusion, the V3-V4 approach enabled us to capture larger alpha-diversities, although the dissimilarity structures across cohorts, in terms of relative abundance of individual taxa, were relatively comparable in both datasets. Other reports have demonstrated that variable 16S rRNA regions can differently impact the taxa detected and thus the overall microbial community structures depicted, the largest differences being detected at lower taxonomic ranks (Bukin et al., 2019). Prior studies have described that V1-V3 regions may capture higher taxonomic diversities than V3-V4 when assessing oral and fecal samples (Zheng et al., 2015). This contrasts with the results observed in the present study with human milk samples, where V3-V4 revealed the highest alpha-diversity indices, which also yielded a higher representation of low abundance groups although at expenses of yielding a higher representation of unclassified minority phyla. However, at this point we cannot conclude whether this is due to a better resolution of this 16S rRNA region in this particular ecological niche, characterized by a relative lower complexity than the human gut, or to differences in the DNA extraction method, the sequencing depth and/or the criteria to exclude or trim the sequences after the quality analysis. For instance, differences in the levels of Proteobacteria or Actinobacteriota levels detected through both approaches, could be the result of differences in the selected primers’ efficiency to amplify those groups, whereas an overall increased alpha diversity in the V3-V4 approach could either be the result of more efficient amplification of unrelated bacteria with selected universal primers and/or of the higher sequencing depth achieved. Although none of the short 16S rRNA hipervariable regions can provide the taxonomic resolution achieved by full length 16S rRNA sequencing, they can still provide meaningful information on the composition and structure of human associated microbial populations, specifically when reaching sufficient sequencing depths.

Remarkably, despite all the methodological differences between the two approaches, both were able to delineate a similar structure for the human milk microbiome of the different cohorts from which samples were collected, in terms of overall most abundant taxa and the differences these presented among cohorts. These results agree with previous results where patterns of predictions compared among different pipeline analysis were comparable provided that the sequencing depth and choice of NGS remain similar (Rajan et al., 2019), and suggest that the main inter-population and intra-population differences previously reported for the milk microbiome of the INSPIRE cohort are genuine as they could be corroborated through an independent different sequencing approach.

In addition, it must be highlighted that new versions of databases may introduce an important bias when trying to compare results within the same laboratory or among different laboratories. As an example, some phyla, such as Actinobacteriota or Patescibacteria, that appear as relevant for human milk microbiome using Silva 138 did not appear as such by using the previous version Silva 132. Discordances at the genus level may also arise, for example in relation to *Rhodanobacter* versus *Dyella* or to *Corynebacterium*_1 versus *Corynebacterium*. Thus, to conduct meaningful comparisons across datasets researchers should consider reanalyzing raw reads through common pipelines and reference databases.

## Data Availability Statement

The datasets generated in this study can be found in the SRA repository (https://www.ncbi.nlm.nih.gov) under Bioproject Accesion Number PRJNA693239.

## Ethics Statement

All study procedures were approved by the overarching Washington State University Institutional Review Board (#13264) and at each study location, consent was obtained from each participating woman. The patients/participants provided their written informed consent to participate in this study.

## Author Contributions

LR, CA, CG-C, EJ, KL, EK-M, EK, and SM conducted the research. MKM, CM, JF, DS, SEM, AP, DG, GO, RP, LB, MAM, JW, and JR designed the research. LR, CA, and JR wrote the manuscript. LR, CA, and JR had primary responsibility for the final content of the manuscript. LR and CA analyzed the data. All authors contributed to the article and approved the submitted version.

## Funding

This project was primarily funded by the National Science Foundation (award 36 #1344288). LR (Spain) was funded by grant 624773 (FP-7-PEOPLE-2013-IEF, European Commission), project AGL2013-4190-P (Ministry of Economy and Competitiveness, Spain), and has received funding from RTI2018-095021-J-I00 (Ministry of Science Innovation and Universities CIU/AEI/FEDER, UE).

## Conflict of Interest

The authors declare that the research was conducted in the absence of any commercial or financial relationships that could be construed as a potential conflict of interest.
