# Supplementary material for: Comparison of Two Approaches for the Metataxonomic Analysis of the Human Milk Microbiome
Source: Front Cell Infect Microbiol. 2021 Mar 25;11:622550. doi: 10.3389/fcimb.2021.622550 (PMC8027255; doi:10.3389/fcimb.2021.622550)

Supplementary Material

Comparison of two approaches for the metataxonomic analysis of the human milk microbiome

Lorena Ruiz[1,†,*], Claudio Alba[1], Cristina García-Carral [1], Esther A Jiménez [1,♯], Kimberly A Lackey[2], Janet E Williams[2], Michelle K McGuire[2], Courtney L Meehan[3], James Foster[2], Daniel W Sellen[4], Elizabeth W Kamau-Mbuthia[5], Egidioh W Kamundia[5], Samwel Mbugua[5], Sophie E Moore[6,7], Andrew M Prentice[8], Debela Gindola K[9], Gloria E Otoo[10], Rossina G Pareja[11], Lars Bode[12], Mark A McGuire[13], Janet E Williams[13], Juan M Rodríguez[1]

[1]Department of Nutrition and Food Science, Complutense University of Madrid, Madrid, Spain.
[2]Margaret Ritchie School of Family and Consumer Sciences, University of Idaho, Moscow, ID, United States
[3]Department of Anthropology, Washington State University, Pullman, WA, United States.
[4]Dalla Lana School of Public Health, University of Toronto, Toronto, ON, Canada.
[5]Department of Human Nutrition, Egerton University, Nakuru, Kenya.
[6]Division of Women's Health, King's College London, London, United Kingdom.
[7]MRC Unit, Serekunda, Gambia.
[8]MRC International Nutrition Group, London School of Hygiene and Tropical Medicine, London, United Kingdom.
[9]Department of Anthropology, Hawassa University, Hawassa, Ethiopia.
[10]Department of Nutrition and Food Science, University of Ghana, Accra, Ghana.
[11]Instituto de Investigación Nutricional, Lima, Peru.
[12]Department of Pediatrics, and Mother Milk Infant Center of Research Excellence (MoMICoRE), University of California, San Diego, La Jolla, CA, United States.
[13]Department of Animal and Veterinary Science, University of Idaho, Moscow, ID, United States.

Current address:
†Instituto de productos Lácteos de Asturias (IPLA-CSIC), Villaviciosa, Asturias, Spain & Instituto de Investigación Sanitaria del Principado de Asturias (ISPA), 33011 Oviedo, Spain.
[♯]Probisearch S.L., C/Santiago Grisolía, Tres Cantos, Spain.

*, corresponding author

KEYWORDS: breast milk, microbiota, 16S rRNA, sequencing reproducibility

Supplementary Table 1. Milk microbial diversity as assessed using the Shannon and Simpson diversity indices. Statistically significance of the comparison among continents is represented by upper case letters; and significance of the comparison among the African, European and American cohorts is represented by lower case letters.

	**Shannon**		**Simpson**	
	**Median (IQR)**	**Significance***	**Median (IQR)**	**Significance***
**AFR**	3.1 (2.41-3.59)	A	0.9 (0.8-0.94)	A
**ETR**	3.48 (2.97-3.79)	a	0.91 (0.87-0.94)	ac
**ETU**	2.59 (1.54-3.49)	ab	0.84 (0.58-0.89)	b
**GBR**	3.06 (2.51-3.48)	ab	0.91 (0.78-0.94)	abc
**GBU**	3.28 (2.97-3.6)	a	0.93 (0.9-0.95)	ab
**GN**	3.17 (1.35-3.8)	ab	0.89 (0.69-0.94)	abc
**KE**	2.85 (2.49-3.18)	b	0.89 (0.8-0.92)	c
**AM**	2.91 (2.44-3.45)	A	0.87 (0.8-0.93)	A
**USC**	2.44 (1.89-2.88)	b	0.83 (0.71-0.88)	c
**USW**	2.99 (2.53-3.52)	ab	0.89 (0.83-0.93)	abc
**PE**	2.95 (2.4-3.55)	ab	0.88 (0.8-0.94)	abc
**EU**	2.83 (2.34-3.5)	A	0.88 (0.79-0.94)	A
**SP**	2.9 (2.34-3.56)	ab	0.88 (0.76-0.94)	abc
**SW**	2.68 (2.42-3.48)	ab	0.88 (0.82-0.92)	abc

*Pairwise comparisons using Wilcoxon rank sum test with Bonferroni adjustment method.

Supplementary Table 2. Pairwise Adonis values obtained for all the comparisons among cohorts.

Pairs	SumsOfSqs	F.Model	R2	p-value	Corrected p-value
**ETR vs ETU**	4.3600829	13.538334	0.16206134	0.000999	0.05
**ETR vs GBR**	4.3572264	13.805755	0.16473517	0.000999	0.05
**ETR vs GBU**	4.437358	14.52154	0.17180875	0.000999	0.05
**ETR vs GN**	4.4019106	12.756405	0.15414402	0.000999	0.05
**ETR vs KE**	5.3452631	18.792074	0.20697923	0.000999	0.05
**ETR vs PE**	4.4592831	15.602207	0.18662434	0.000999	0.05
**ETR vs SP**	4.1862003	13.120198	0.15784609	0.000999	0.05
**ETR vs SW**	4.1051062	15.120272	0.23218994	0.000999	0.05
**ETR vs USC**	2.9892473	9.734709	0.16574031	0.000999	0.05
**ETR vs USW**	5.7528506	20.928867	0.22766371	0.000999	0.05
**ETU vs GBR**	0.891372	2.472755	0.03072785	0.001998	0.05
**ETU vs GBU**	1.1121474	3.16429	0.03898623	0.000999	0.05
**ETU vs GN**	1.0557508	2.7286	0.03379967	0.000999	0.05
**ETU vs KE**	0.830582	2.506999	0.03038528	0.001998	0.05
**ETU vs PE**	1.634942	4.880477	0.06034184	0.000999	0.05
**ETU vs SP**	0.8380745	2.30507	0.02870391	0.000999	0.05
**ETU vs SW**	0.8467273	2.505697	0.04141257	0.003996	0.05
**ETU vs USC**	0.5212755	1.41012	0.0241417	0.10589411	0.25
**ETU vs USW**	1.2486645	3.862233	0.0466103	0.000999	0.054
**GBR vs GBU**	0.5102758	1.476133	0.01857329	0.04195804	0.17
**GBR vs GN**	1.2316884	3.231622	0.03978281	0.000999	0.05
**GBR vs KE**	0.7063056	2.168805	0.0263945	0.001998	0.05
**GBR vs PE**	1.0697911	3.251052	0.04102219	0.001998	0.05
**GBR vs SP**	0.8421333	2.353678	0.02929147	0.000999	0.05
**GBR vs SW**	1.1957618	3.621959	0.05877709	0.000999	0.05
**GBR vs USC**	0.835007	2.308228	0.03891918	0.002997	0.05
**GBR vs USW**	1.5000278	4.723155	0.05641396	0.000999	0.05
**GBU vs GN**	1.3250761	3.560815	0.04365841	0.000999	0.05
**GBU vs KE**	0.8050965	2.540683	0.03078098	0.001998	0.05
**GBU vs PE**	1.1637248	3.638757	0.04569078	0.000999	0.05
**GBU vs SP**	0.9026535	2.58799	0.03211384	0.000999	0.05
**GBU vs SW**	1.2734817	4.004324	0.06458137	0.000999	0.05
**GBU vs USC**	1.0307351	2.94981	0.04920466	0.000999	0.05
**GBU vs USW**	1.5734201	5.097002	0.0606086	0.000999	0.05
**GN vs KE**	1.8402777	5.236277	0.06143249	0.000999	0.05
**GN vs PE**	2.1500983	6.036229	0.07358004	0.000999	0.05
**GN vs SP**	1.3934638	3.626567	0.04442876	0.000999	0.05

**GN vs SW**	1.6545522	4.524298	0.07236064	0.000999	0.05
**GN vs USC**	1.0873396	2.732437	0.04574461	0.000999	0.05
**GN vs USW**	2.2866085	6.652947	0.0776733	0.000999	0.05
**KE vs PE**	1.3479174	4.493873	0.05447524	0.000999	0.05
**KE vs SP**	1.014466	3.086394	0.03714681	0.000999	0.05
**KE vs SW**	0.8365511	2.862377	0.04553403	0.000999	0.05
**KE vs USC**	0.8786397	2.72739	0.04418444	0.001998	0.05
**KE vs USW**	1.0830311	3.736686	0.04409762	0.000999	0.05
**PE vs SP**	0.8795389	2.647275	0.0336601	0.000999	0.05
**PE vs SW**	1.3652786	4.636274	0.0764604	0.000999	0.05
**PE vs USC**	0.8483104	2.597488	0.04509724	0.001998	0.05
**PE vs USW**	1.8110062	6.21642	0.07470184	0.000999	0.05
**SP vs SW**	0.8801397	2.63268	0.04342015	0.000999	0.05
**SP vs USC**	0.6309826	1.724012	0.02935788	0.02597403	0.05
**SP vs USW**	1.0793655	3.366154	0.04086817	0.000999	0.05
**SW vs USC**	0.4797644	1.466797	0.03813151	0.08391608	0.25
**SW vs USW**	0.3233369	1.151164	0.01913785	0.25174825	0.25
**USC vs USW**	0.7779463	2.500679	0.04133308	0.001998	0.05

Supplementary Table 3. 16S rRNA region, number of samples and number of high-quality reads in the milk samples analyzed either with the V3-V4 (this work) or the V1-V3 (Lackey et al., 2019) approach.

Region	Samples	N Seq	Min	Median (IQR)	Max
V3_V4	392	18945135	7317	38902.5 (28200-57088.5)	431772
V1_V3	394	7516849	1391	13033.5 (8353.5-22062)	141620
Total	786	26461984	1391	26233 (12990.75-43024.5)	431772

Supplementary Figure 1

Flowchart depicting the disposition of the milk samples included in the study of Lackey et al. (2019) and in this study. ETR, rural Ethiopia; ETU, urban Ethiopia; GBR, rural Gambia; GBU, urban Gambia; GN, Ghana; KE, Kenya; SP, Spain; SW, Sweden; PE, Peru; USC, California (United States); USW, Washington (United States).

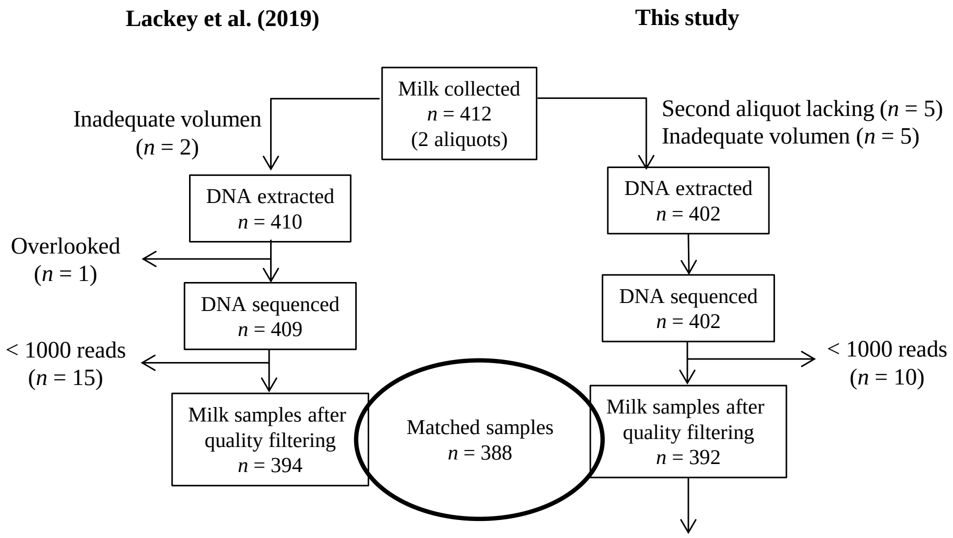

**Cohort**	**Samples (*n*)**
**AFRICA**	234
**ETR**	32
**ETU**	40
**GBR**	40
**GBU**	40
**GN**	40
**KE**	42
**AMERICA**	98
**PE**	38
**USC**	19
**USW**	41
**EUROPE**	60
**SP**	40
**SW**	20
**Total**	392

Supplementary Figure 2

Boxplot of the 10 most abundant genera found in each location with the SILVA 138 database, including the groups of unclassified genera and minor genera.

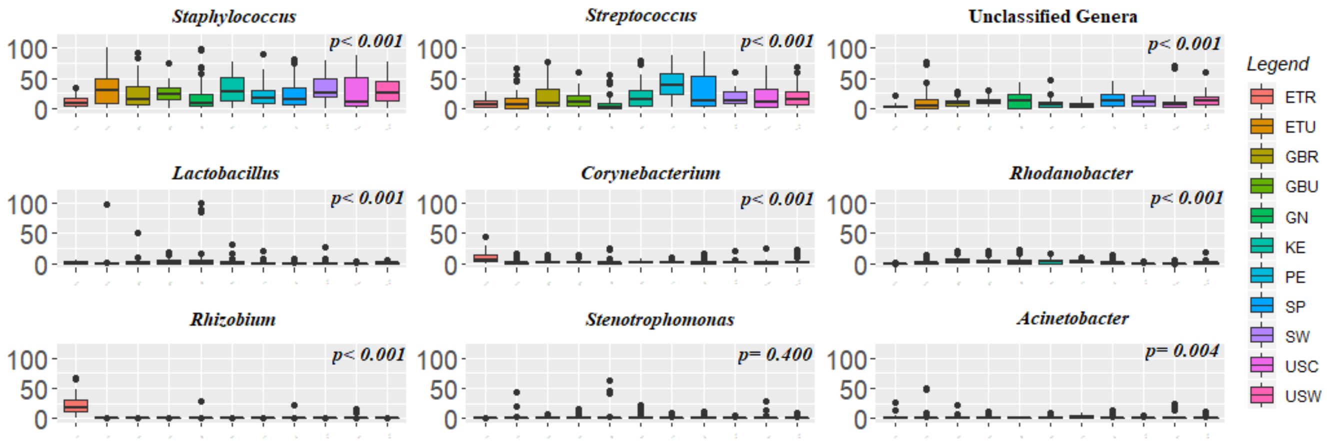

Supplement: Supplementary file 1 [file DataSheet_1.pdf]
